# Measuring the acceptability of EQ-5D-3L health states for different ages: a new adaptive survey methodology

**DOI:** 10.1007/s10198-021-01424-8

**Published:** 2022-01-05

**Authors:** Zoltán Hermann, Márta Péntek, László Gulácsi, Irén Anna Kopcsóné Németh, Zsombor Zrubka

**Affiliations:** 1 Institute of Economics, Centre for Economic and Regional Studies, Tóth Kálmán u 4., Budapest, 1097 Hungary; 2 Institute of Economics, Corvinus University of Budapest, Fővám tér 8., Budapest, 1093 Hungary; 3 Health Economics Research Center, Óbuda University, Bécsi út 96/b, Budapest, 1034 Hungary; 4 Corvinus Institute for Advanced Studies, Corvinus University of Budapest, Fővám tér 8., Budapest, 1093 Hungary; 5 Department of Infection Control, Medical Centre, Hungarian Defence Forces, Róbert Károly körút 44., Budapeset, 1134 Hungary

**Keywords:** Acceptability, EQ-5D, Societal preferences, Priority setting, Sufficientarianism, I10

## Abstract

**Background:**

Acceptable health and sufficientarianism are emerging concepts in health resource allocation. We defined acceptability as the proportion of the general population who consider a health state acceptable for a given age. Previous studies surveyed the acceptability of health problems separately per EQ-5D-3L domain, while the acceptability of health states with co-occurring problems was barely explored.

**Objective:**

To quantify the acceptability of 243 EQ-5D-3L health states for six ages from 30 to 80 years: 1458 health state–age combinations (HAcs), denoted as the acceptability set of EQ-5D-3L.

**Methods:**

In 2019, an online representative survey was conducted in the Hungarian general population. We developed a novel adaptive survey algorithm and a matching statistical measurement model. The acceptability of problems was evaluated separately per EQ-5D-3L domain, followed by joint evaluation of up to 15 HAcs. The selection of HAcs depended on respondents’ previous responses. We used an empirical Bayes measurement model to estimate the full acceptability set.

**Results:**

1375 respondents (female: 50.7%) were included with mean (SD) age of 46.7 (14.6) years. We demonstrated that single problems that were acceptable separately for a given age were less acceptable when co-occurring jointly (*p* < 0.001). For 30 years of age, EQ-5D-3L health states of ‘11112’ (11.9%) and ‘33333’ (1%), while for 80 years of age ‘21111’ (93.3%) and ‘33333’ (7.4%) had highest and lowest acceptability (% of population), respectively.

**Conclusion:**

The acceptability set of EQ-5D-3L quantifies societal preferences concerning age and disease severity. Its measurement profiles and potential role in health resource allocation needs further exploration.

**Supplementary Information:**

The online version contains supplementary material available at 10.1007/s10198-021-01424-8.

## Background and aims

The quality-adjusted life-year (QALY), by combining both the quality and length of life in a single figure became a key measure of health gains in health economic analyses [1]. For the measurement of the quality-of-life component of QALYs, the EQ-5D instrument is preferred in many countries [2]. EQ-5D describes distinct health states, to which societal preferences (utility scores) are attached to quantify quality-of-life [3, 4]. Utility scores are elicited in valuation studies via methods that are rooted in multi-attribute utility theory, such as time-trade-off or discrete choice, which involve choices between different durations spent in full health or various disease states [2–5].

A salient feature of QALYs is their measurement invariance concerning the severity of disease and age, described by the catchy phrase “a QALY is a QALY is a QALY” [6]. However, in case of similar QALY gains, in scarce-resource settings, people prefer to treat more severe patients over less severe ones and young adults over older ones [7, 8]. Despite their simplicity and widespread use [9], QALYs do not reflect adequately these and several other preferences that matter in decision-making [9, 10]. To overcome these limitations, numerous improvements and alternative frameworks have been proposed [11, 12].

The normative background for using acceptable health in resource allocation has been explored by Wouters et al. [13], building on the sufficientarian theory of distributive justice [14]. The concept of acceptable health is based on the finding that people consider certain health problems increasingly acceptable for older ages as a normal consequence of aging [15–17]. The main idea is that the treatment of individuals in not acceptable health would enjoy priority over treating those who are in acceptable health states, while the goal would be to ensure acceptable (but not necessarily perfect) health for all [13, 18].

Acceptability and utility scores are both theoretically and quantitatively different measures of health. Although not based on standard economic theory, acceptability has been used as a measurable rating for a complex set of subjective judgements [19], such as the overall “goodness” of a health state. Measuring acceptability via binary yes/no questions carries as much information about a population’s judgements as continuous measures [20]. As opposed to valuation studies [4], the evaluation of acceptability does not involve choices concerning risk, no trade-off as well as no imaginations about death or different time perspectives are involved. However, instead of attaching a single utility value to a health state, acceptability of a health state is measured in different ages. Throughout this paper, the term “acceptable health” will refer to the general concept, while “acceptability” will denote a measure: the proportion of the general population, who consider a certain health state or problem acceptable for (people in) a given age. We also note that acceptability of a health state is conceptually different from the acceptability of a health intervention [21].

Acceptable health has been measured via the EQ-5D-3L instrument in several studies [15–17, 22]. EQ-5D-3L describes three levels of problems in five health domains [3]. Although EQ-5D-3L has gradually been replaced by the five-level (EQ-5D-5L) version due to its more favourable psychometric properties [23], immense experience has been gained with EQ-5D-3L in general population surveys and clinical studies [24, 25] and its 243 health states are better suited for the evaluation of acceptability than the 3125 health states described by EQ-5D-5L.

Using traditional survey methods, the direct measurement of the acceptability of all EQ-5D-3L health states for several ages would require prohibitively large samples or long questionnaires. Therefore, initial studies assessed the acceptability of problems separately in each of the five health domains. Via this method, the acceptability of all EQ-5D-3L health states could only be deduced if assuming that either (1) joint problems in multiple health domains were not acceptable at all or (2) separately acceptable problems were also acceptable when co-occurring jointly. However, these two assumptions led to rather divergent results, so the acceptability for all EQ-5D-3L health states could not be estimated accurately so far [15–17].

The primary aim of this paper is to measure the acceptability of all 243 EQ-5D-3L health states at 30, 40, 50, 60, 70 and 80 years of age in the Hungarian general population and develop an acceptability set for EQ-5D-3L. To estimate the acceptability of health states with problems in multiple health domains, we have developed an adaptive survey methodology and a matching statistical measurement model and tested whether this method delivers more accurate acceptability estimates compared to the assumption that separately acceptable problems are also acceptable when co-occurring jointly.

## Methods

### Data

We performed a cross-sectional online survey in May 2020 using quotas proportional to the ≥ 18-year-old general population in terms of age, gender, education and geographical region. We planned to recruit 1200 respondents. Participation was voluntary and anonymous, and participants gave their written informed consent prior completing the questionnaire. Our study was approved by the Ethical Committee of the Medical Research Council of Hungary (ETT TUKEB; 3857-5-2019/EKU). Data were collected by a market survey company, no compensation was given for participating in the study.

### Measuring acceptability

**Fig. 1:**
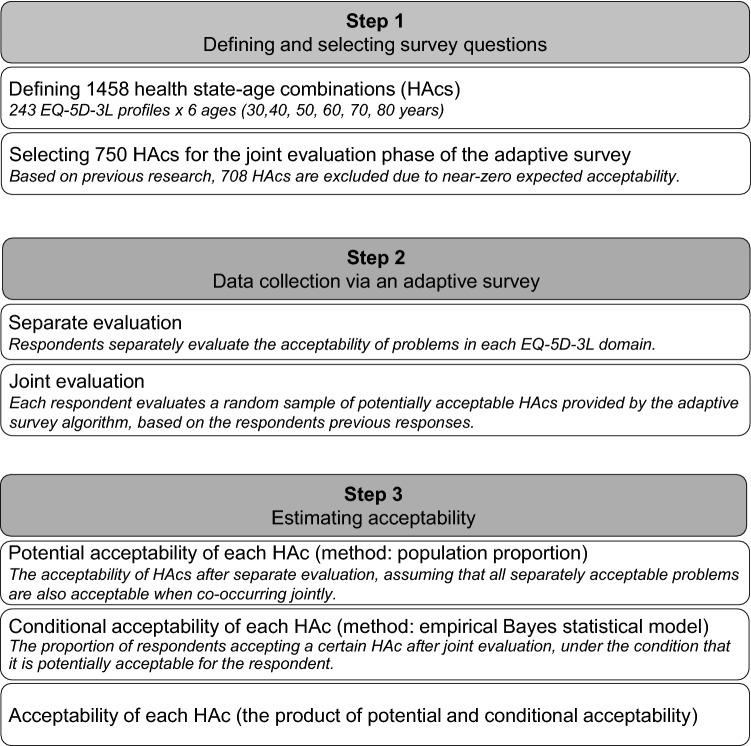
The process of measuring acceptability

Measuring the acceptability of health states with problems in multiple health domains is a stepwise process that involves (1) the selection of potential survey questions and (2) conducting an adaptive survey to boost the information content of collected data and then (3) estimating acceptability via a statistical measurement model that mitigates the bias resulting from the adaptive survey design. The key steps of this method are summarized in Fig. 1.

### Selecting potential survey questions

We evaluated acceptability using the EQ-5D-3L instrument [3]. The descriptive system of EQ-5D-3L assesses self-reported health in five domains: mobility, self-care, usual activities, pain/discomfort and anxiety/depression. In each dimension, respondents can describe their health as having: no problems (1), some problems (2) or severe problems (3), providing 243 (3^5^) distinct health states [3]. EQ-5D-3L health states are denoted with 5-digit numbers indicating the problem levels in the five domains (e.g., 21,131 represents moderate problems with mobility and severe pain/discomfort). The EQ-5D-3L index is a utility value attached to a health state that reflects average preferences of the general population so that 1 denotes perfect health, 0 denotes death and negative values denote worse-than-dead health states. A value set comprises the index values of all 243 health states. To compare our results with previous studies, we applied the Dutch value set [16, 26].

To measure acceptability, we estimated the proportion of respondents who considered an EQ-5D-3L health state acceptable for ages 30, 40, 50, 60, 70 and 80 years. We will denote a health state – age combination (HAc) with a subscript of age attached to the EQ-5D-3L health state (e.g., 12113_50_). Altogether, we used 1458 HAcs (243 EQ-5D-3L health states × 6 ages) and denoted the full set of acceptability estimates attached to them as the EQ-5D-3L acceptability set. While we defined the acceptability of a HAc as a proportion, in case of a given respondent, acceptability of a HAc will refer to the result of a binary yes / no evaluation.

From the 1458 HAcs, we preselected 750 items with multiple health problems for joint evaluation. We will denote these HAcs as the JE frame. By narrowing the question pool to the JE frame, we aimed to increase the precision of acceptability estimates. Also, respondents were allocated to predefined random question sequences of the JE frame, which allowed the mitigation of bias that resulted from the adaptive survey design (see below). The JE frame excluded 642 HAcs, that were almost universally rated as not acceptable in previous research [17], 60 HAcs that contain problems in only one domain and 6 HAcs denoting full health (see Online Resource 1). Although not all HAcs were included in the JE frame, the acceptability was estimated for all 1458 HAcs.

### Acceptability survey questions and the adaptive survey algorithm

**Fig. 2:**
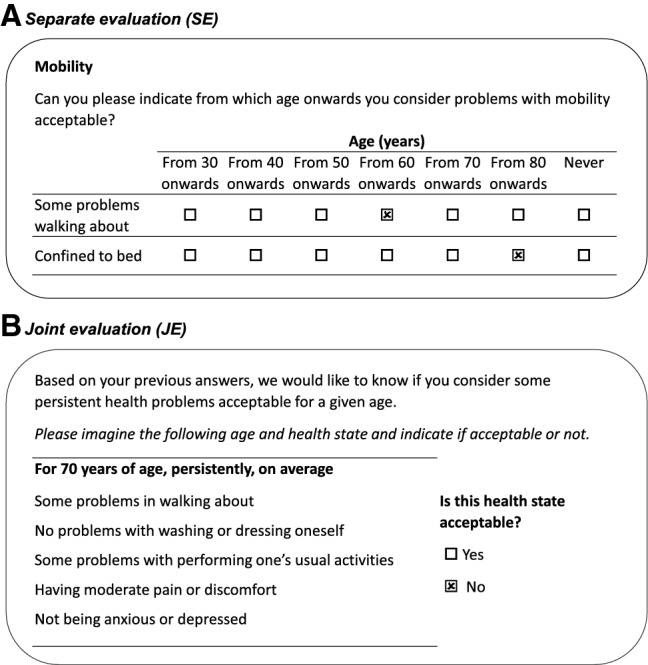
Sample questions of the adaptive survey

The acceptability survey comprised two stages. First, respondents were asked from what age onwards they considered moderate or severe problems acceptable in each EQ-5D domain. The response options were 30, 40, 50, 60, 70 and 80 years of age or never. The sample question is depicted in Fig. 2A. Previous studies evaluated acceptability using the same question format, albeit the age range varied [15, 16, 22, 27]. As the acceptability of health problems was evaluated separately per EQ-5D-3L domain, we will refer to this part of the survey as separate evaluation (SE).

In the second stage of the survey, respondents evaluated HAcs with multiple problems as either acceptable or not acceptable (Fig. 2B). Depending on the answers in SE, up to 15 semi-random questions were selected by an adaptive survey algorithm. Since the acceptability of co-occurring health problems in HAcs was evaluated jointly by respondents, this part of the survey is denoted as joint evaluation (JE).

The idea of the adaptive survey algorithm is that due to the ordinal structure of EQ-5D-3L response levels, by knowing the acceptability of a HAc, the acceptability of numerous other HAcs can be deduced for a given respondent, narrowing the set of questions that can contain additional information for the elicitation of his or her preferences.

This deduction builds on two main assumptions. The first assumption is consistency: as EQ-5D-3L dimensions are ordinal measures or health, if a health state is acceptable, then all health states that contain only the same or lower levels of health problems (denoted as better health states) are also assumed to be acceptable for a given age. If a health state is not acceptable, then all health states that contain only the same or greater levels of problems (worse health states) are not acceptable either for a given age. However, no inferences can be made about two health states that contain both higher and lower problem levels in any domains of the EQ-5D-3L. The second assumption is monotonicity in age: if a health state is acceptable for a certain age, then we consider the same or better health states acceptable for older ages as well. At the same time, if a health state is not acceptable for a certain age, inferences can be made about the non-acceptability of the same or worse health states for younger ages.

After the SE stage of the survey, 60 HAcs with a single health problem (e.g., 21111_60_) could be classified as acceptable or unacceptable for each respondent. The remaining HAcs with multiple health problems could be labelled as either unacceptable or potentially acceptable using the assumptions above. Those HAcs, which contained unacceptable problem levels in any domain could be categorised as not acceptable. However, the joint acceptability of co-occurring problems remained unknown for those HAcs (e.g., 21122_60,_ 21121_60_, 21112_60_ or 11122_60_), which included combinations of problems that were acceptable one by one during SE (e.g., 21111_60_, 11121_60_, 11112_60_)_._ We denoted these HAcs as potentially acceptable. Only potentially acceptable HAcs were subject to joint evaluation.

The set of potentially acceptable HAcs varies depending on respondents’ preferences, but it is generally too large for an all-encompassing evaluation in a survey situation. The adaptive survey algorithm aims to maximise the obtained information about the unique preference profile of respondents using no more than 15 JE questions per respondent, while maintaining a structure that allows unbiased acceptability estimation for each HAc. The following paragraphs introduce the main steps of the JE procedure. Details are provided in the Online Resource 2.

First, one of the 50 predefined HAc sequences of the JE frame was allocated to the respondent. The actual JE questions of the respondent were selected from the potentially acceptable HAcs of the JE frame. Starting with the first potentially acceptable HAc in the sequence, the respondent was asked to evaluate it. Then the algorithm moved to the next potentially acceptable HAc, and its acceptability was either deduced from prior responses (indirect evaluation) or the respondent was subsequently asked to evaluate it directly. The algorithm stopped when the respondent had answered 15 questions or all potentially acceptable HAcs had been evaluated via less than 15 questions.

Altogether, by moving along the predefined sequence, each respondent directly or indirectly evaluated a subset of *k* HAcs, which we denote as the JE response set. The JE response set is a random sample of potentially acceptable HAcs evaluated as acceptable or not acceptable, with a sample size varying by each respondent.

### Statistical measurement model

As the number of jointly evaluated HAcs depends on respondents’ preferences, the sheer proportion of respondents who accept a HAc would lead to biased acceptability estimates (see Online Resource 3). Therefore, we estimate acceptability using a statistical measurement model, which mitigates bias and provides acceptability estimates for HAcs that were not included in the JE frame.

First, we decompose the acceptability (A^j^) of a given HAc (denoted as HAc^j^, such as 12123_50_) into the product of its potential acceptability (PA^j^) and conditional acceptability (CA^j^) as shown in Eq. (1). PA^j^ refers to the proportion of the population who consider each health problem of HAc^j^ separately acceptable for the given age. CA^j^ denotes the estimated proportion of respondents who jointly evaluate HAc^j^ as acceptable, given that HAc^j^ is potentially acceptable for them. CA^j^ is estimated from the JE response set, since HAc^j^ is evaluated by a given respondent only if it is potentially acceptable after SE.

1 $${\varvec{A}}^{{\varvec{j}}} = PA^{j} \times CA^{j}$$

The two terms are estimated using two different methods. From the complete dataset after SE (1458 HAcs for all respondents), the first term, PA is estimated directly as the proportion of respondents potentially accepting the given HAc. Estimates are adjusted by post-stratification weights to correct for sampling error (see below).

CA is estimated from an incomplete dataset, since not all potentially acceptable HAcs can be evaluated via 15 questions by all respondents in JE. Moreover, JE responses are unevenly distributed across HAcs. Those HAcs, which are potentially acceptable for many respondents (e.g., mild problems in older ages), have plenty observations, while other HAcs with low potential acceptability (e.g., severe problems in younger ages) receive only few or even zero JE responses. To minimize prediction error in this unbalanced data structure, CA^j^ is estimated using an empirical Bayes strategy by combining the direct acceptability estimates and regression model-based parametric estimates. To reduce the mean square error of prediction, the empirical Bayes or shrinkage approach optimally balances the measurement error of direct estimates of CA^j^ from the JE response set of each respondent and model error of parametric estimates of CA^j^ from the combined JE response of all respondents [28, 29]. For technical details see Online Resource 3.

CA^j^ is estimated by weighted ordinary least squares (OLS) regression, where weights are the products of two components: (1) a population weight (post-stratification weights to correct for sampling error) and (2) an information weight to correct for the bias arising from the unbalanced data structure of JE responses. We compare two models and select the one with better fit based on Akaike’s information criteria (AIC) [30], Schwarz’s Bayesian information criteria BIC [31] and likelihood ratio test results. Model 1 (M_1_) is specified as the one used for estimating UK time-trade-off utility values in the MVH study: the predictors include moderate and severe problem levels in each EQ-5D-3L domain and an N3 term for the presence of any severe problems [7]. In addition, the predictors of Model 2 (M2) include dummy variables denoting different levels of PA. For technical details, see Online Resource 4.

As a final step, we calculate acceptability (*A*^*j*^) for each HAc according to Eq. (1). The exceptions are 60 HAcs with a single health problem (e.g., 21111_30_, 21111_40_…11113_80_). These HAcs are not evaluated jointly, and their acceptability is estimated from SE responses as a population proportion like PA. Furthermore, by definition, the acceptability of full health (11111_30–80_) is 1 for any age.

### Auxiliary analyses

In addition to quantifying an acceptability set for EQ-5D-3L, we also performed auxiliary analyses.

#### Descriptive statistics

We applied unweighted descriptive methods to summarize sample characteristics and components of the statistical measurement model. The association between acceptability and PA as well as acceptability profiles of selected HAcs over age were shown graphically.

#### Assessment of data quality

As a signal of respondent effort, we measured response time during the JE task and excluded respondents whose mean response time per question was too short (≤ 8 s) to comprehensively read questions before answering. Details are provided in the Online Resource 5.

Since the JE frame was established using PA estimates of external research [17], to verify its applicability, we calculated the absolute agreement between HAcs included and excluded from the JE frame and those 750 HAcs with multiple problems, which had greatest and 708 HAcs with lowest PA measured in our study.

In EQ-5D-3L valuation studies, logically inconsistent responses (i.e., valuation results that contradict the logical order of health states) were explored and included in the estimation samples in varying proportions [32–36]. However, indirect evaluation in JE automatically provides all possible logically consistent answers, so responses to direct questions cannot be inconsistent, not even from a “random” responder. Therefore, to assess the “truthfulness” of answers, respondents directly evaluated 5 fixed HAcs after JE as control questions and we calculated the absolute agreement between responses to JE questions (direct and indirect evaluations) and the control questions.

#### Comparing results with previous research

JE was applied first in this study, but given the similar sampling strategies, we compared our SE results with those of the Netherlands [16] as follows. Prior studies summarised SE results by assuming that separately acceptable problems were also acceptable when co-occurring jointly. For each respondent, the highest levels of acceptable problems for the six ages were aggregated as acceptable HAcs. The sample mean of the EQ-5D-3L index scores of these HAcs in each age was denoted as the aggregate acceptable health curve (AHC_aggregate_) [15–17]. We graphically compare the AHC_aggregate_ of our sample with that of the Netherlands using Dutch EQ-5D-3L index values [26].

Finally, we formally tested the hypothesis whether adding CA estimates in the statistical measurement model (and conducting JE) improves the accuracy of acceptability estimates compared to using PA estimates (and conducting SE) alone. According to Eq. (1), if separately acceptable problems are also acceptable when co-occurring jointly, then CA^j^ = 1 for all j. We tested the assumption that CA^j^ = 1 via Wald test and tested whether CA^j^ is a constant across all HAcs via the overall likelihood ratio test of the parametric estimation model of CA^j^ (see above). All analyses were performed in Stata 16 statistical software package [37].

## Results

### Sample characteristics

Recruitment was extended to achieve low education quotas, so 1453 individuals provided answers in the survey. Mean (SD) response time per question in JE was 41 (189) seconds, median response time was 21 s. Based on adequate response times, we included 1375 (94.6%) respondents in the analysis sample (hereinafter: sample). Mean (SD) age was 46.7 (14.6) years. The sample was similar to the general population in terms of gender and region, while the 65 + age group and lower education group was under- and the 50–64 age group and the higher education group was overrepresented (Table 1).

**Table 1:** Sample demographic characteristics

		Sample		Survey		Population
		*N*	%	*N*	%	%
Gender	Women	678	49.31	724	49.83	53.18
	Men	697	50.69	729	50.17	46.82
Age	18–34	350	25.45	384	26.43	23.25
	35–49	405	29.45	438	30.14	28.96
	50–64	444	32.29	454	31.25	24.69
	65 +	176	12.80	177	12.18	23.10
Education	Lower secondary or below (ISCED0-2)	179	13.02	187	12.87	23.21
	Upper secondary: vocational (ISCED3c)	223	16.22	240	16.52	21.89
	Upper secondary: general (ISCED3a,4)	476	34.62	500	34.41	33.14
	Tertiary (ISCED5-6)	497	36.15	526	36.20	21.76
Region	Central Hungary	405	29.45	430	29.59	30.92
	Northern Hungary	162	11.78	173	11.91	11.42
	Northern Great Plain	208	15.13	217	14.93	14.59
	Southern Great Plain	184	13.38	193	13.28	12.89
	Western Transdanubia	132	9.60	137	9.43	10.14
	Central Transdanubia	133	9.67	141	9.70	10.83
	Southern Transdanubia	151	10.98	162	11.15	9.20
Total		1375	100.00	1453	100.00	100.00

Source of population data: Micro-census 2016 [46]

### The components of acceptability

#### Acceptability of problems in separate evaluation

**Fig. 3:**
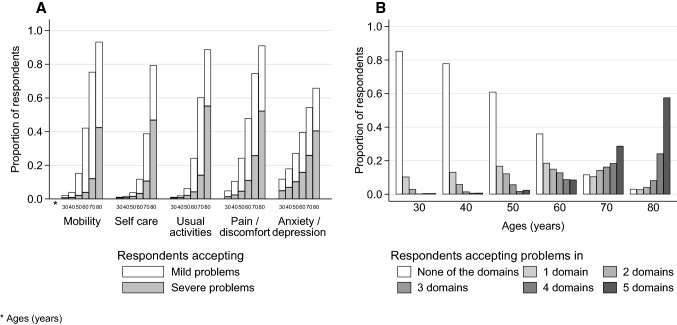
The proportion of respondents in separate evaluation (SE) who accept problems **A** by EQ-5D domain and age **B** by the number domains and age

The acceptability of problems increased steeply beyond 50 years of age in all EQ-5D-3L domains. The acceptability of problems in the anxiety / depression domain was slightly less age dependent, and self-care problems were less acceptable for 60 and 70 years of age than problems in other domains (Fig. 3A).

The number of domains with acceptable problems increased with age. For age 30, 85.3% (1173/1375), 10.4% (143/1375) and 0.5% (7/1375), while for age 80, 3.1% (42/1375), 2.4% (38/1375) and 57.6% (792/1375) of respondents considered problems in none, only one and all five EQ-5D-3L domains acceptable, respectively (Fig. 3B). The preferences of respondents were rather heterogenous in SE. Among 1375 respondents we identified 1029 different patterns of acceptable health problems.

#### Verifying the JE frame

Online Resource 6 provides the distribution of potential acceptability for two subsets of HAcs. Fig. S5A depicts the distribution for those 642 HAcs that contained multiple problems but were excluded from the JE frame. Their median (range) potential acceptability was 0.006 (0.003–0.035). Fig. S5B depicts the distribution of potential acceptability for the 750 HAcs included in the JE frame. Their median (range) potential acceptability was 0.100 (0.009–0.880). According to potential acceptability, 96.3% of HAcs with multiple problems (1340/1392) were allocated correctly into or out of the JE frame. The threshold separating the top 750 HAcs in terms of PA was > 0.019 in our sample.

#### Results of joint evaluation

Those 1295 respondents participated in JE, who considered multiple problems acceptable for a given age during SE. Out of 189,346 potentially acceptable HAcs, 38,174 (20.2%) were evaluated during JE, including 14,585 (38.2%) direct evaluations and 23,589 (61.8%) indirect evaluations. The average JE response set contained 11.3 direct evaluations (median: 15, range: 1–15) and 18.2 indirect evaluations (median: 11, range: 0–458). Altogether 694/1375 (50.4%) respondents performed 15 direct evaluations. On average, from each respondent, 29.5 (direct and indirect) evaluations (median: 25, range: 0–468) were included in the JE response set. From the 750 HAcs of the JE frame, 695 (92.7%) were evaluated in JE.

#### Estimates of conditional acceptability

The empirical Bayes estimates of CA^j^ included both the direct- and parametric estimate components for those 329 HAcs of the JE frame, which received 15 or more (direct or indirect) joint evaluations and altogether 95.1% (36,292/38,174) of JE responses. The CA^j^ for those 421 HAcs, which had < 15 joint evaluations were estimated only via parametric methods.

Table 2 presents the weighted OLS models that provide the parametric component of empirical Bayes estimates of CA^j^. Due to superior fit, M_2_ was chosen for estimating the acceptability set. The significant overall likelihood ratio test confirmed that CA^j^ is not constant across all HAcs. The coefficients of M_2_ are interpreted as follows. Like the results of SE (Fig. 3A), problems in the anxiety / depression domain affected conditional acceptability differently than problems in other domains. While the coefficients for both moderate and severe problems were significant in other domains, the presence of anxiety / depression had marked effect on conditional acceptability without significant difference between severe and moderate problem levels. The presence of any severe problems (N3 term) was not significant, while lower levels of potential acceptability were associated with lower conditional acceptability.

**Table 2:** Regression model of conditional acceptability

		M_1_	M_2_
Moderate problems	Mobility	– 0.0234*** (0.0071)	– 0.0246*** (0.0071)
	Self-care	– 0.0272*** (0.0088)	– 0.0248*** (0.0090)
	Usual activities	– 0.0278*** (0.0080)	– 0.0295*** (0.0080)
	Pain / discomfort	– 0.0281*** (0.0082)	– 0.0289*** (0.0081)
	Anxiety / depression	– 0.0628*** (0.0097)	– 0.0529*** (0.0102)
Severe problems	Mobility	– 0.1090*** (0.0179)	– 0.1000*** (0.0179)
	Self-care	– 0.0646*** (0.0193)	– 0.0635*** (0.0198)
	Usual activities	– 0.0587*** (0.0174)	– 0.0597*** (0.0174)
	Pain / discomfort	– 0.0631*** (0.0175)	– 0.0587*** (0.0172)
	Anxiety / depression	0.0228 (0.0178)	0.0188 (0.0181)
Any severe problem (N3)	Yes	– 0.0425*** (0.0116)	– 0.0182 (0.0142)
Potential acceptability (PA)	0–0.05		-0.1010*
			(0.0544)
	0.05–0.1		-0.0535
			(0.0340)
	0.1–0.2		-0.0858***
			(0.0224)
	0.2–0.3		-0.0544***
			(0.0189)
	0.3–0.4		-0.0544***
			(0.0151)
	0.4–0.5		-0.0390***
			(0.0144)
	0.5–0.6		-0.0009
			(0.0114)
Constant		0.983***	0.9980***
		(0.0140)	(0.0138)
Observations		38,174	38,174
R-squared		0.081	0.084
AIC		42,925.66	42,853.5
BIC		43,028.26	43,015.9
LR-test chi2			86.2
Prob > chi2			< 0.001

Notes: Lineal probability model estimates. Dependent variable: HAc is acceptable in JE. Observations: respondent-HAc. Robust standard errors clustered at the individual level in parentheses. Base level for potential acceptability: Range: 0.6–1

^***^p < 0.01, **p < 0.05, *p < 0.1

#### Evaluating the consistency of responses

Due to the varying size of JE response sets, out of the 5 control questions, 5, 4, 3, 2, 1 and 0 could be evaluated by 1009 (73.4%), 204 (14.8%), 108 (7.9%), 40 (2.9%), 10 (0.7%) and 4 (0.3%) individuals from the 1375 respondents, respectively. From the 1371 respondents who answered 1–5 control questions, only 304 (22.2%) provided fully consistent answers, while no more than one inconsistent answer was provided by 636 (46.4%). Altogether, 91.3% of the control questions were answered and 61.0% of the responses to control questions matched JE responses. JE response time was not an indicator of inconsistent answers. The proportion of consistent answers did not differ between the analysis sample and those respondents, who were excluded due to short JE answer times (OR 0.994, *p* = 0.947).

#### The EQ-5D-3L acceptability set

The estimated EQ-5D-3L acceptability set for the 1458 HAcs is presented in Table S2 of Online Resource 7. For 30, 40, 50, 60, 70 and 80 years of age the most acceptable HAcs and their acceptability were 11112_30_ (0.119), 11112_40_ (0.179), 11112_50_ (0.272), 11121_60_ (0.479), 21111_70_ (0.754) and 21111_80_ (0.933), respectively. The acceptability of 33333_30_, 33333_40,_ 33333_50,_ 33333_60,_ 33333_70_ and 33333_80_ was 0.001, 0.001, 0.002, 0.006, 0.021 and 0.074, respectively.

**Fig. 4:**
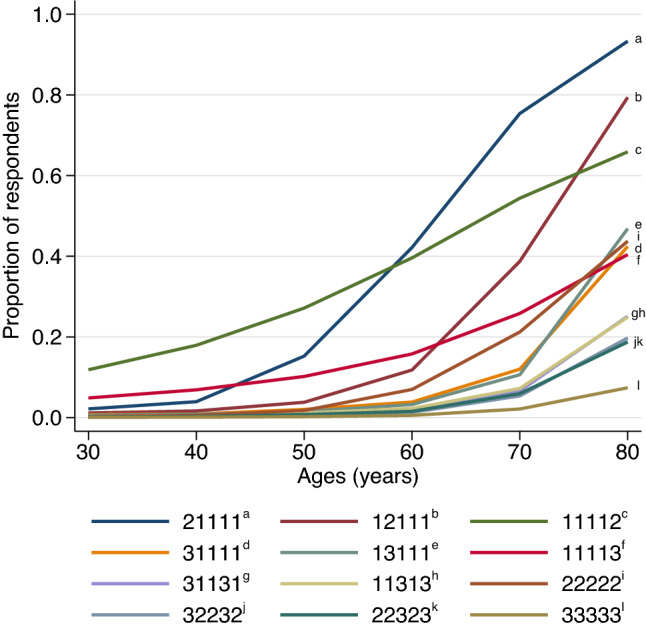
Acceptability profiles of selected health states-age combinations (HAcs)

Figure 4 displays the acceptability profiles of 12 selected health states over age. The acceptability profiles of single-problem health states were shaped by the EQ-5D-3L domain and problem severity. However, the acceptability profiles of health states with multiple problems seemed to depend mainly on the number and severity of problems, and not on the affected domain.

### Comparing results with previous research

#### Comparing Hungarian and Dutch results of separate evaluation

**Fig. 5:**
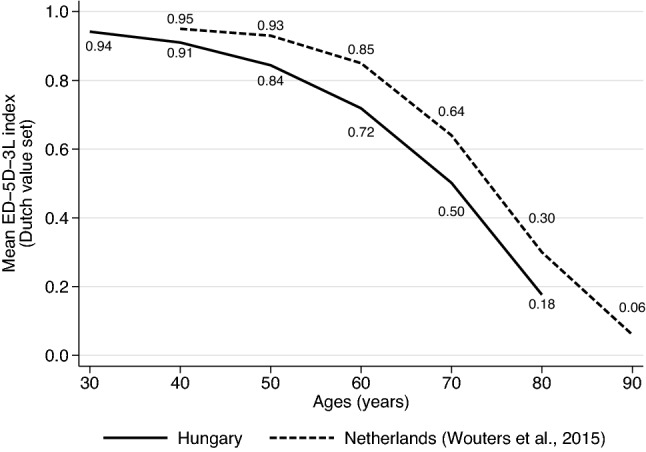
Aggregate acceptable health curves (AHC_aggregate_) of Hungary and the Netherlands

Figure 5 depicts the AHC_aggregate_ of our sample with that of the Netherlands. Despite the similar shape of both curves, the Dutch AHC_aggregate_ is shifted to the right suggesting that similar levels of health problems are considered acceptable for 5–10 years older ages in the Netherlands than in Hungary. The EQ-5D-3L index differences between the two curves ranged between 0.04 and 0.14 with the greatest difference at 70 years of age. Higher AHC_aggregate_ values denote higher EQ-5D-3L index values (less problems) acceptable for a given age.

#### Comparing the accuracy of the adaptive algorithm versus separate evaluation

The overall weighted proportion of positive JE responses in our sample was 0.732 (SE: 0.012). We rejected the hypothesis that this proportion (CA) is equal to 1 (F_1, 1294_ = 481.33, p < 0.001), confirming that separately acceptable problems are not universally acceptable when co-occurring jointly.

**Fig. 6:**
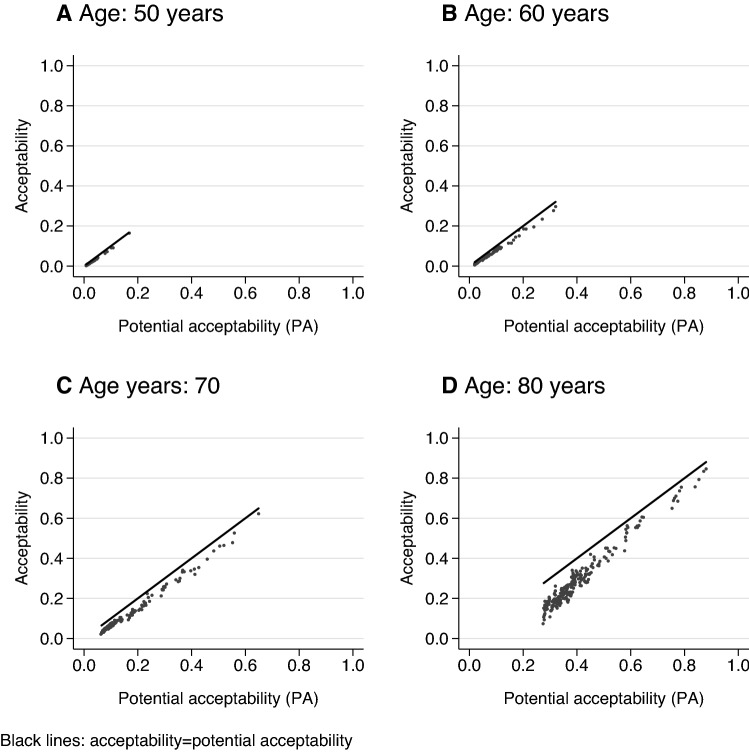
Association of acceptability and potential acceptability of HAcs with multiple problems over different ages

Figure 6 illustrates the resulting differences between acceptability and potential acceptability in case of 1392 HAcs with multiple problems. Although most respondents accepted co-occurring health problems jointly as well, acceptability was lower than PA for most HAcs, especially at ages over 50 years. The difference increased with the number and severity of health problems (represented by lower levels of PA).

## Discussion

### Main findings

In this cross-sectional survey, for 1458 HAcs (243 EQ-5D-3L health states in six ages from 30 to 80 years), we quantified the proportion of the general population who consider them acceptable: an acceptability set for EQ-5D-3L. We estimated acceptability via a novel adaptive survey involving joint evaluation of co-occurring problems, followed by a matching statistical measurement model. Using this method, we have shown that from those potentially acceptable HAcs that contained multiple problems, less than ¾ were acceptable in joint evaluation, depending on the age as well as the number and severity of jointly occurring health problems of HAcs.

### Elaboration of results

Acceptability has previously been measured via SE both in the general population [15–17] and chronic patients [27]. Compared to that of the Hungarian population, higher AHC_aggregate_ values of the Netherlands may signal higher health standards corresponding with higher life expectancy and generally healthier lifestyle [38, 39]. AHC_aggregate_ values are also available from a non-representative sample of the Hungarian general population [17] and a preliminary version of the adaptive survey was tested in a small pilot study on a convenience sample [18]. Since the perception of acceptable health depends on individual characteristics of respondents, such as age, socio-economic and health status [40], more nationally representative surveys are needed to demonstrate that acceptability is a stable and reliable measure of population-level health preferences.

The strength of our study is that we determined the complete acceptability set for EQ-5D-3L in 6 ages from 30 to 80 years, which, unlike EQ-5D-3L value sets, quantifies societal preferences regarding age and the severity of disease in a structured and transparent way. The questions were designed to elicit respondents’ judgements about HAcs “concerning people in general” for the six ages and not concerning themselves, reflecting the oft preferred perspective for reimbursement decisions [41]. By the secondary use of previously collected EQ-5D-3L data, the acceptability set allows the exploration of new health outcome measures motivated by sufficientarian theory of distributive justice. Sufficientarians assert that once individuals have secured enough, the reason to further benefit them changes. Acceptability offers a natural threshold which allows the application of different weights to acceptable versus not acceptable health states (e.g., acceptable life years, or QALY gains in unacceptable health states), which may provide a plausible equity weighting scheme that reflects societal preferences concerning age and severity of disease. The acceptability set allows the estimation of acceptability of health states as a function of utility and age, leading to a straightforward implementation in usual decision-model structures [11, 13, 42, 43]. However, the role of acceptable health in healthcare resource allocation, its link to positive and negative utilities as well as the state of death requires further exploration.

## Limitations

A limitation of our study is that despite our efforts to verify main steps of the estimation process and assess data quality, many aspects of the measurement properties of the acceptability set and its sensitivity to alternative methodological choices remained unexplored and require future research. We designed a statistical measurement model to mitigate bias arising from the dependence of survey questions on respondents’ preferences, yet its statistical properties need to be explored in greater depth. Although the rate of inconsistent answers to control questions was similar to the rate of logically inconsistent answers in EQ-5D-3L valuation studies [33, 34], the exclusion criteria due to low quality of responses have not yet been established for acceptability. Based on our preliminary exploration, neither the answer times, not the control questions provided a fully reliable basis for the exclusion of potentially inconsistent respondents. For example, providing purely “yes” or “no” answers in JE may both signal strategic responses or a respondent’s true preferences. Both may result in fast response times and congruent answers to control questions. Therefore, we chose to exclude respondents sparingly, only due to too brief response times to read and comprehend HAc vignettes in JE. Also, in some countries, the EQ-5D-3L population norms report flat or decreasing prevalence of problems in the anxiety / depression domain, where the assumption of monotonicity in age may have to be relaxed [24]. However, the steep increase of anxiety / depression problems with age in Hungary suggests that the assumptions of the adaptive algorithm represented adequately the experiences of the general population [24].

Another limitation was that our survey was conducted in an online population, which typically under-represents individuals with lower education or older age groups [44]. However, the Canadian EQ-5D-3L valuation study is an example that online surveys may be adequate means for health preference research [4] and the adaptive algorithm can be applied in computer-aided personal interviews [18, 45].

## Conclusion

In conclusion, we quantified an acceptability set for EQ-5D-3L using a novel adaptive survey algorithm and a matching statistical measurement model, which provided more accurate estimates than prior methods. However, in-depth understanding of the statistical and psychometric properties of the new method requires further research, and the potential role of acceptability in health decision-making needs to be explored.

## Supplementary Information

Below is the link to the electronic supplementary material.

Supplementary file1 (DOCX 125 KB)

Supplementary file2 (DOCX 256 KB)

Supplementary file3 (DOCX 124 KB)

Supplementary file4 (DOCX 131 KB)

Supplementary file5 (DOCX 361 KB)

Supplementary file6 (DOCX 143 KB)

Supplementary file7 (DOCX 144 KB)

## Data Availability

Data are available upon reasonable request from the authors.

## Code availability

The code of statistical analyses is available upon reasonable request from the authors.
