# Supplementary material for: Measuring the acceptability of EQ-5D-3L health states for different ages: a new adaptive survey methodology
Source: Eur J Health Econ. 2022 Jan 5;23(7):1243–55. doi: 10.1007/s10198-021-01424-8 (PMC9395309; doi:10.1007/s10198-021-01424-8)
Supplement: Supplementary file 1 — Supplementary file1 (DOCX 125 KB) [file 10198_2021_1424_MOESM1_ESM.docx]

**Online Resource 1**

***Selecting 750 health state-age combinations (HAcs) for joint evaluation (JE frame)***

The JE frame was determined from separate evaluation (SE) results of large sample of the Hungarian general population (N=9260) [17].

We set a problem-score (PS) constructed from the sum of the 4^th^ power of EQ-5D-3L problem levels in each domain. This score ranked HAcs with moderate problems first, giving a score of 5 for 11111_30-80_, score 20 for HAcs with a single moderate problem (e.g., 21111_30-80,_ 12111_30-80_), score 80 for HAcs with five moderate problems (22222_30-80_), and score 85 for HAcs with at least one severe problem (e.g., 31111_30-80,_ 13111_30-80_). The highest PS was 405 (33333_30-80_). We excluded from the JE frame 66 HAcs containing a single problem, for which acceptability could be determined from SE results (PS=20 and PS=85). We also excluded HAcs with PS levels, for which mean potential acceptability was <0.003 (Table S1). From 1392 HAcs with multiple problems, 681 had potential acceptability >0.003.

Furthermore, we excluded HAcs from the JE frame that contained problem dimensions, which were associated with potential acceptability <0.003. Hence, we excluded HAcs including moderate mobility problems (potential acceptability=0.0017), moderate self-care problems (potential acceptability=0.0011) and moderate problems with usual activities (potential acceptability=0.0021) at age 30, moderate self-care problems (potential acceptability=0.0018) at age 40 and severe self-care problems at age 50 (potential acceptability=0.0014).

Finally, we added two outlier health-states that had extremely high potential acceptability within excluded PS groups. For 11123_30_ and 11123_40_, potential acceptability was 0.0084 and 0.0193, respectively. Altogether, in ages 30, 40, 50, 60, 70 and 80 years, 2, 7, 66, 211, 232 and 232 HAcs were included in the JE frame, respectively.

**Table S1 Potential acceptability of HAcs by problem score and age**

| **N of moderate problems** | **N of severe problems** | **Problem score** | **Age (years)** | | | | | |
| --- | --- | --- | --- | --- | --- | --- | --- | --- |
|  |  |  | **30** | **40** | **50** | **60** | **70** | **80** |
| 2 | 0 | 35 | 0.0033 | 0.0084 | 0.0318 | 0.1225 | 0.3993 | 0.7372 |
| 3 | 0 | 50 | 0.0014 | 0.0027 | 0.0125 | 0.0680 | 0.3034 | 0.6547 |
| 4 | 0 | 65 | 0.0012 | 0.0019 | 0.0067 | 0.0421 | 0.2420 | 0.5865 |
| 5 | 0 | 80 | 0.0011 | 0.0016 | 0.0041 | 0.0278 | 0.1987 | 0.5284 |
| 1 | 1 | 100 | 0.0014 | 0.0025 | 0.0069 | 0.0235 | 0.0945 | 0.3449 |
| 2 | 1 | 115 | 0.0008 | 0.0013 | 0.0034 | 0.0158 | 0.0839 | 0.3251 |
| 3 | 1 | 130 | 0.0008 | 0.0011 | 0.0024 | 0.0115 | 0.0757 | 0.3071 |
| 4 | 1 | 145 | 0.0008 | 0.0011 | 0.0019 | 0.0089 | 0.0691 | 0.2904 |
| 0 | 2 | 165 | 0.0010 | 0.0015 | 0.0028 | 0.0083 | 0.0443 | 0.2445 |
| 1 | 2 | 180 | 0.0007 | 0.0011 | 0.0017 | 0.0062 | 0.0414 | 0.2343 |
| 2 | 2 | 195 | 0.0007 | 0.0010 | 0.0015 | 0.0051 | 0.0390 | 0.2247 |
| 3 | 2 | 210 | 0.0007 | 0.0010 | 0.0014 | 0.0044 | 0.0370 | 0.2156 |
| 0 | 3 | 245 | 0.0007 | 0.0010 | 0.0013 | 0.0033 | 0.0264 | 0.1936 |
| 1 | 3 | 260 | 0.0007 | 0.0010 | 0.0012 | 0.0030 | 0.0257 | 0.1870 |
| 2 | 3 | 275 | 0.0007 | 0.0010 | 0.0012 | 0.0028 | 0.0250 | 0.1806 |
| 0 | 4 | 325 | 0.0006 | 0.0010 | 0.0012 | 0.0024 | 0.0205 | 0.1656 |
| 1 | 4 | 340 | 0.0006 | 0.0010 | 0.0012 | 0.0024 | 0.0202 | 0.1605 |
| 0 | 5 | 405 | 0.0006 | 0.0010 | 0.0012 | 0.0022 | 0.0178 | 0.1472 |

Sample N=9260, source data: Péntek et al. [17]
