# Supplementary material for: Measuring the acceptability of EQ-5D-3L health states for different ages: a new adaptive survey methodology
Source: Eur J Health Econ. 2022 Jan 5;23(7):1243–55. doi: 10.1007/s10198-021-01424-8 (PMC9395309; doi:10.1007/s10198-021-01424-8)
Supplement: Supplementary file 2 — Supplementary file2 (DOCX 256 KB) [file 10198_2021_1424_MOESM2_ESM.docx]

**Online Resource 2**

***Technical details of the adaptive survey algorithm***

In the following we provide technical details about

1. Creating pre-defined joint evaluation (JE) question sequences from the JE frame
2. The JE procedure for an individual respondent
3. ***Creating pre-defined JE question sequences***

The 750 health state age combinations (HAcs) of the JE frame were split into 15 strata by potential acceptability and age, as shown in Fig S1. Each stratum contained 50 HAcs. We created 50 question sequences from the JE frame by cycling through the 15 consecutive strata and selecting HAcs randomly without replacement. Thereby all 750 HAcs were scattered evenly by potential acceptability and age across the sequence.

**Fig S1 Splitting the JE frame into 15 HAc strata by potential acceptability and age**

1. ***The JE procedure for an individual respondent***

Respondents were allocated consecutively to one of the 50 predefined HAc sequences. After separate evaluation (SE), only potentially acceptable HAcs were kept in the sequence for each respondent, preserving the predefined order. Starting with the first potentially acceptable HAc in the sequence, respondents performed direct evaluation. The term direct evaluation of a HAc denotes the actual response to a JE question by a respondent, while indirect evaluation denotes the automatic deduction about the acceptability of a HAc based on the main assumptions of consistency and monotonicity in age.

The algorithm subsequently moved to the next potentially acceptable HAc in the sequence and performed indirect evaluation. If the HAc could be evaluated indirectly, the algorithm moved to the next potentially acceptable HAc. If the HAc could not be evaluated indirectly, the respondent was asked to perform direct evaluation. Indirect evaluation is illustrated by the following example: if 22122_70_ was directly evaluated as acceptable from a HAc sequence of 22122_70,_ 22122_80,_ 22121_70_, 21123_60_ and 11223_60_ then 22122_80_ and 22121_70_ could also be evaluated as acceptable indirectly. However, the acceptability of 21123_60_ and 11223_60_ could not be deduced from previous responses, so the algorithm asked direct evaluation. JE continued until either 15 HAcs had been directly evaluated, or the joint acceptability of all potentially acceptable HAcs could be directly or indirectly evaluated. Altogether, by moving along the predefined sequence, each respondent jointly evaluated a subset of *k* HAcs, which we denote as the JE response set.

The steps of the JE procedure are illustrated schematically in Fig S2.

***Step 0:*** one of the 50 question sequences of the JE frame is allocated randomly to the respondent. The different colours denote the 15 HAc strata from which the HAcs were randomly selected

***Step 1:*** after separate evaluation (SE) potentially acceptable HAcs are selected from the JE frame.

***Step 2:*** the respondent starts with direct evaluation of the 1^st^ HAc in the sequence

***Step 3:*** the algorithm moves to the 2^nd^ HAc in the sequence. In this example this HAc is indirectly evaluated as acceptable, based on the results of the previous direct evaluation.

***Step 4:*** the algorithm moves to the next HAc in the sequence, which cannot be evaluated indirectly, therefore the 2^nd^ direct evaluation is performed. In this example this HAc is evaluated as unacceptable.

***Steps 5 and 6***: the next two HAcs can be evaluated indirectly as not acceptable, based on previous responses.

***Step 7-9:*** these HAcs cannot be evaluated indirectly, so the algorithm asks for the 3^rd^, 4^th^ and 5^th^ direct evaluation.

***Step k:*** the algorithm stops at the 15^th^ direct evaluation at the *k^th^* HAc. All preceding HAcs in the sequence are evaluated as acceptable or unacceptable. These *k* HAcs are denoted as the JE response set (denoted with black (acceptable) and white (not acceptable) colours. In this example 7 potentially acceptable HAcs were not evaluated.

**Fig S2 Schematic illustration of the JE procedure**

**
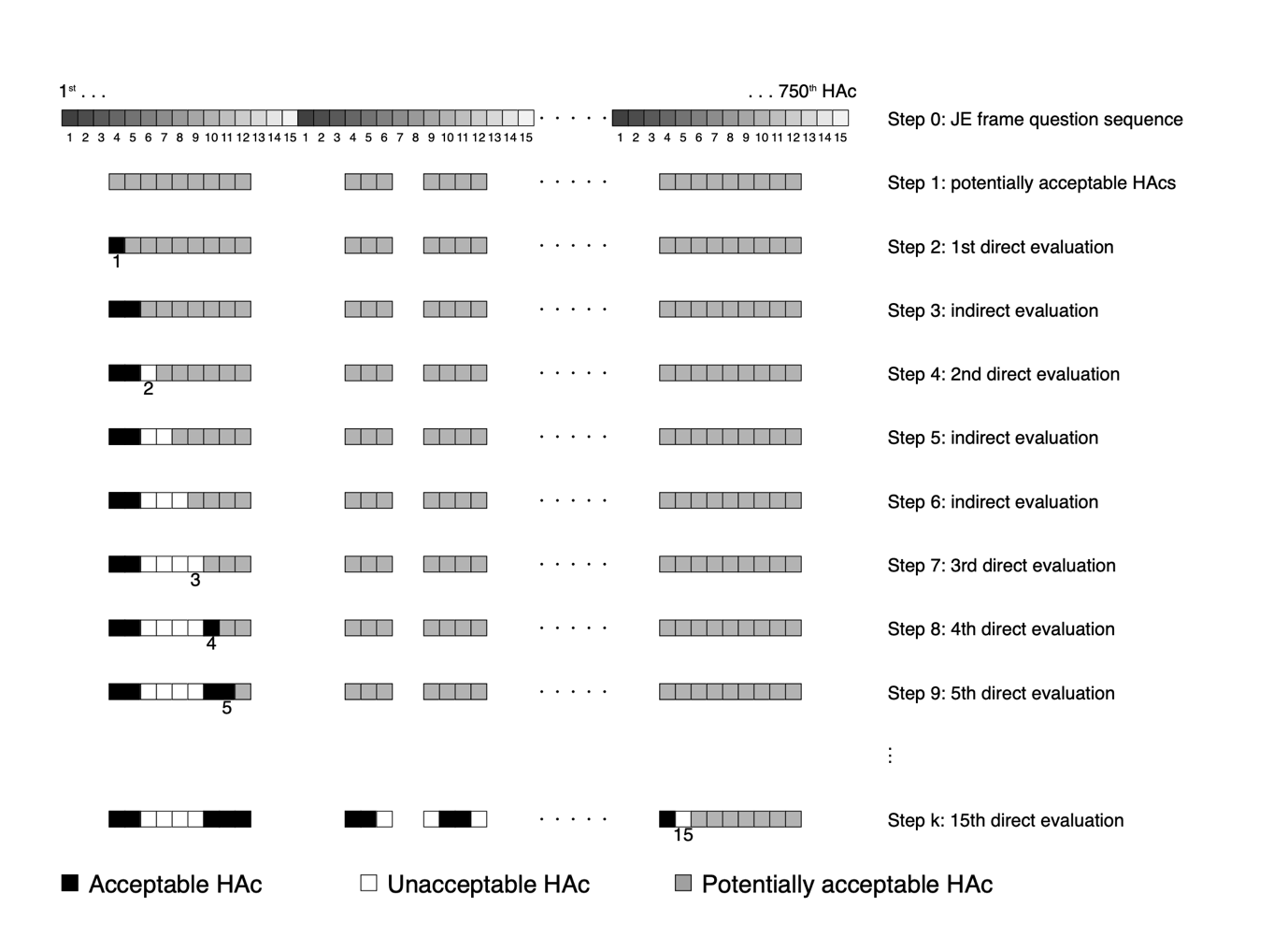
**
