# Supplementary material for: Measuring the acceptability of EQ-5D-3L health states for different ages: a new adaptive survey methodology
Source: Eur J Health Econ. 2022 Jan 5;23(7):1243–55. doi: 10.1007/s10198-021-01424-8 (PMC9395309; doi:10.1007/s10198-021-01424-8)
Supplement: Supplementary file 3 — Supplementary file3 (DOCX 124 KB) [file 10198_2021_1424_MOESM3_ESM.docx]

**Online Resource 3**

***The empirical Bayes estimates of conditional acceptability***

For each HAc*^j^,* the empirical Bayes estimates of *CA^j^* were calculated as a precision weighted average of the following two terms

| $\boldsymbol{CA}^{\boldsymbol{j}}\boldsymbol{=}{\hat{\boldsymbol{CA}}}^{\boldsymbol{j}}\boldsymbol{+}\boldsymbol{s}^{\boldsymbol{j}}\boldsymbol{(}{\bar{\boldsymbol{CA}}}^{\boldsymbol{j}}\boldsymbol{-}{\hat{\boldsymbol{CA}}}^{\boldsymbol{j}}\boldsymbol{)}$, | (2) |
| --- | --- |

where $\hat{CA}^{j}$ is the parametric estimate of conditional acceptability, the term *(*$\bar{CA}^{j}- \hat{CA}^{j}$*)* is denoted as the HAc specific component, where $\bar{CA}^{j}$ is the direct estimate of conditional acceptability for HAc^j^ (the weighted mean of the available indirect and direct evaluations for HAc^j^*)* and *s^j^* is the shrinkage factor representing the relative precision of the estimate of the HAc specific component. The less precise the estimate of the HAc specific component (e.g., due to the small number of observations), the closer *s^j^* is to zero and the more CA^j^ is shrunk towards the parametric estimate, attenuating measurement error. At the same time, for HAcs with many JE responses, s^j^ converges to 1. In such cases CA^j^ may deviate recognizably from the parametric estimate $\hat{CA}^{j}$, reflecting the unique preferences for certain HAcs.

We modelled conditional acceptability using a linear probability model as a function of HAc characteristics:

| ${CA}_{i}^{j}=\alpha+\sum\beta X^{j}+\sum\gamma{DPA}^{j}+\varepsilon_{i}^{j}$, | (3) |
| --- | --- |

where X is a vector of 5-5 dummy variables indicating moderate- severe problems in the five domains of EQ-5D and an additional dummy variable indicating the presence of at least one severe health problem, following the specification of the widely used UK EQ-5D-3L value set [7]. To handle potential non-linear effects, potential acceptability was included as a set of dummy variables (DPA^j^) representing the level of the potential acceptability of HAc^j^. We specified model 1 (M_1_) excluding, and model 2 (M_2_) including the DPA component, and evaluated model fit using the Akaike information criterion (AIC) and Bayesian information criterion (BIC) values [30,46] and likelihood ratio test. The final set of acceptability estimates are based on the extended (M_2_) specification.

If the number of JE responses was ≥ 15 for HAc^j^, ${CA}^{j}$was estimated by eq (2) using parametric estimates ($\hat{CA}^{j})$ from eq (3). If 0-14 JE responses were available for HAc^j^ (scarce observations due to low potential acceptability or the HAc was not included in the JE frame) CA^j^ was estimated only by using the parametric estimates ($\hat{CA}^{j})$ from eq (3).

We estimate the shrinkage factor as:

| $s^{j}= \frac{V}{V+V^{j}}$, | (4) |
| --- | --- |

where $V$ is the between variance of HAc specific components across all HAcs and *V^j^* is the variance of the HAc specific component *j*.

We estimate the HAc specific components by regressing the residuals from the model of eq (3) on the set of dummy variables denoting the HAcs:

| $\varepsilon_{i}^{j}= \theta^{j}+\mu_{i}^{j}.$ | (5) |
| --- | --- |

θ^j^ is the fixed effect of HAc^j^, which is identical to the difference between the direct and parametric estimates of conditional acceptability for HAc^j^ in eq (3) or the HAc specific component in eq (2).

Building on the results from eq (3) *s^j^* is estimated for each HAc^j^. Eq (3) provides standard errors for each *θ^j^,* and following [47] we use the squares of these to estimate *V^j^*, the variance of HAc specific component estimates in eq (5). V is estimated as the variance of the θs.

The precision weight (shrinkage factor) of the HAc specific component in the empirical Bayes estimates was estimated for 329 HAcs (having 15 or more observations) according to eq (3) from the residuals of M_2_. The mean (SD) shrinkage factor (s^j^) estimate was 0.60 (0.18), in the range between 0.17-0.98.
