# Supplementary material for: Measuring the acceptability of EQ-5D-3L health states for different ages: a new adaptive survey methodology
Source: Eur J Health Econ. 2022 Jan 5;23(7):1243–55. doi: 10.1007/s10198-021-01424-8 (PMC9395309; doi:10.1007/s10198-021-01424-8)
Supplement: Supplementary file 4 — Supplementary file4 (DOCX 131 KB) [file 10198_2021_1424_MOESM4_ESM.docx]

**Online Resource 4**

***Calculation of weights***

*Population weights*

For the *w^p^* component we used post-stratification weights with respect to 24 strata defined by gender, education and age based on the 2016 Microcensus data of Hungary [45].

*Information weights*

For unbiased estimates of the conditional acceptability of HAc^j^, one would need to observe JE responses from a representative sample of those respondents, who consider HAc^j^ potentially acceptable. However, as the size of the JE response set varied over respondents, the probability of a certain respondent being observed in case of a given HAc also varied. As response rates and preferences were not independent, without correction, this would lead to biased estimates of conditional acceptability. We employed information weights to mitigate this bias.

The more HAcs a respondent evaluated in his/her JE response set, the more likely he/she was included in the direct estimate of conditional acceptability and with the more HAcs he/she contributed to the parametric estimates of conditional acceptability of a given HAc^j^. As the extent of the JE response set is not independent of preferences, without correction, conditional acceptability estimates of HAc^j^ may be biased, which is illustrated by the following example: respondent “A” accepts only mild problems in SE and enters JE with a few potentially acceptable HSs, out of which all will be evaluated and included in his/her JE response set. Respondent “B” accepts mild and severe problems in SE and enters JE with hundreds of potentially acceptable HAcs, out of which a smaller fraction will be evaluated and included in his/her JE response set. Since mild and severe HAcs are scattered randomly in the JE frame, both severe and mild problems will be omitted from the JE response set of “B”. Therefore, while respondent “A” and “B” contribute equally to the potential acceptability estimates, “B” will be underrepresented in the conditional acceptability estimate for mild HSs.

To correct for this bias, we calculated the information weight *w^i^* as follows. Respondents were assigned to three groups based on the proportion of the JE response set compared to the potentially acceptable HAcs within the JE frame. In *w^i^* group 1 respondents evaluated directly or indirectly <0.25 of the potentially acceptable HSs, in *w^i^* group 2 the proportion was 0.25-0.9, while in *w^i^* group 3 the proportion of evaluated HAcs was >0.9. The *w^i^* component was calculated as the inverse of the mean share of JE responses within each group, and then scaled to match the number of JE observations.

In the information weight groups 1, 2 and 3, the number of respondents (and the corresponding information weights) were 492 (2.355), 192 (0.493) and 611 (0.243), respectively.
