# Supplementary material for: Measuring the acceptability of EQ-5D-3L health states for different ages: a new adaptive survey methodology
Source: Eur J Health Econ. 2022 Jan 5;23(7):1243–55. doi: 10.1007/s10198-021-01424-8 (PMC9395309; doi:10.1007/s10198-021-01424-8)
Supplement: Supplementary file 5 — Supplementary file5 (DOCX 361 KB) [file 10198_2021_1424_MOESM5_ESM.docx]

**Online Resource 5**

***Quality assessment based on response times***

We explored the association of preferences (measured as the probability of yes answers) and mean response time in joint evaluation (JE) via locally weighted scatterplot smoothing [48] (Fig. S3). Despite a slight breakpoint noticeable at 8s mean response time, we found no evidence of notably different preference patterns across different response times.

**Fig S3 Probability of yes (acceptable) answers by mean response time in joint evaluation**

**
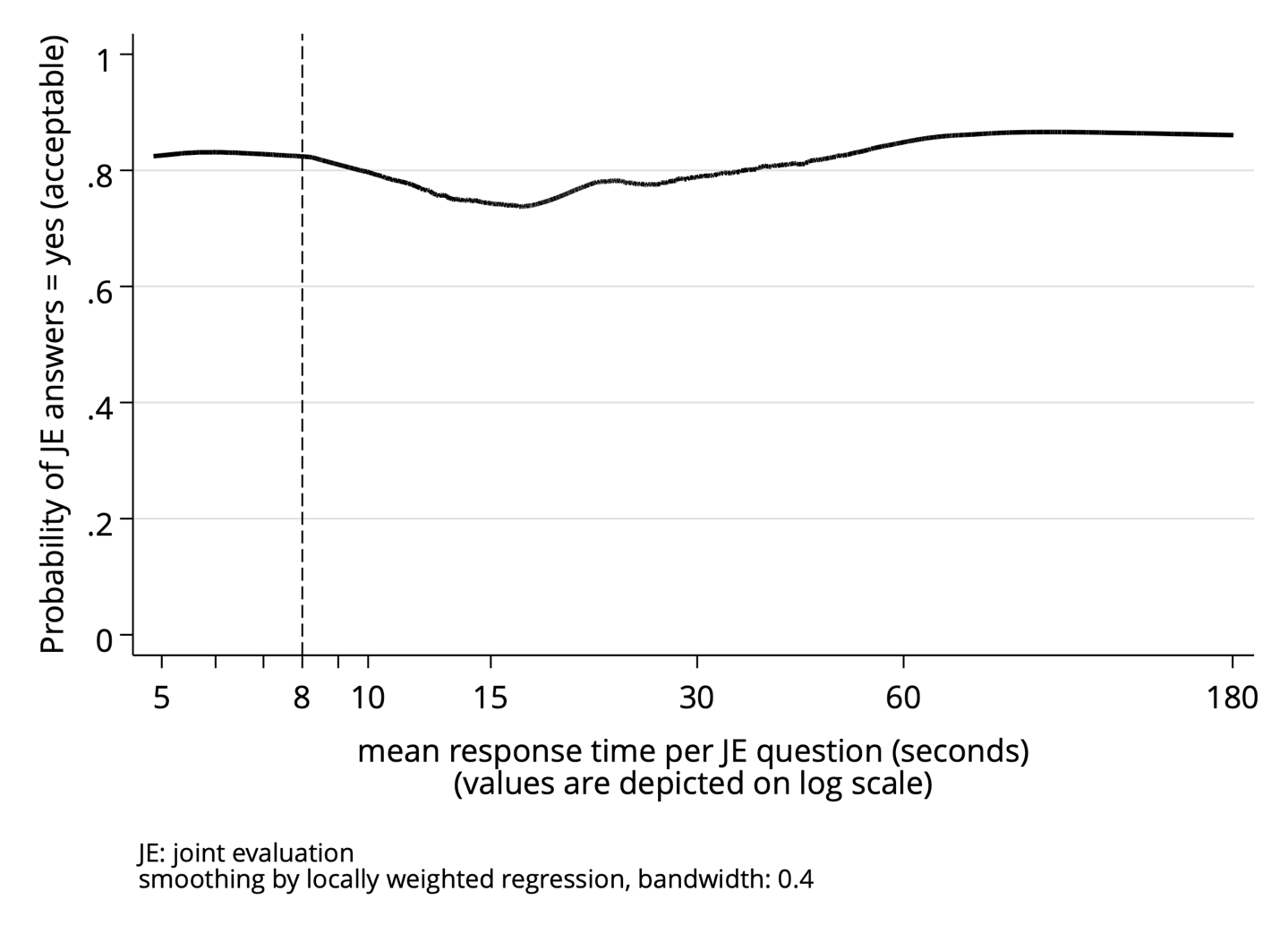
**

We hypothesized that “all yes” answers in JE may be a signal of low respondent effort. Therefore, we also explored the association of “all yes” answers with mean response time in JE via locally weighted scatterplot smoothing (Fig. S4). Although a breakpoint was noticeable at 8s response time, and we observed a convex pattern, there was no evidence for a markedly different preference pattern at short response times.

**Fig S4 Probability of yes (acceptable) answers for all questions by mean response time in joint evaluation**

***
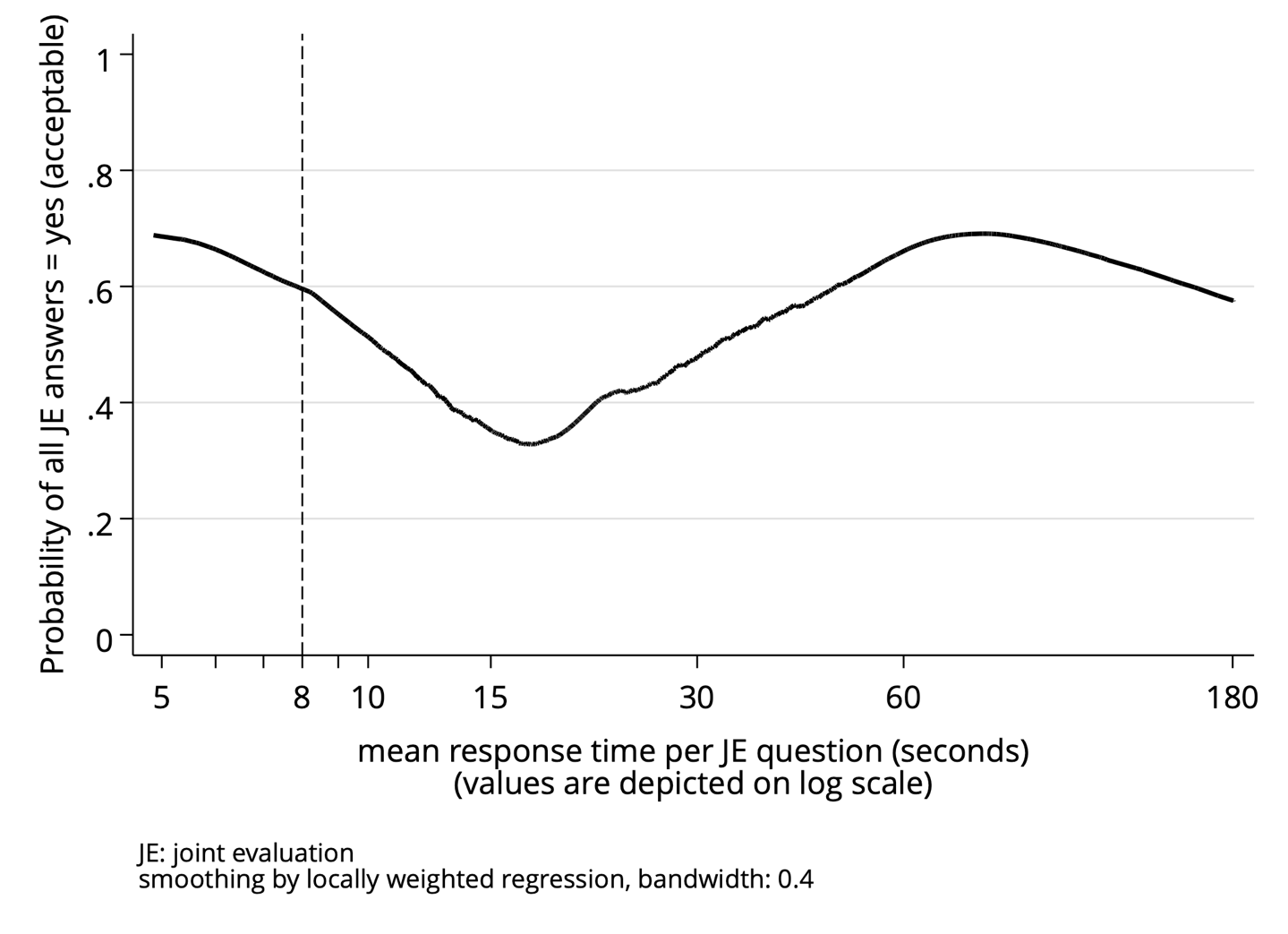
***
