# Supplementary material for: Measuring the acceptability of EQ-5D-3L health states for different ages: a new adaptive survey methodology
Source: Eur J Health Econ. 2022 Jan 5;23(7):1243–55. doi: 10.1007/s10198-021-01424-8 (PMC9395309; doi:10.1007/s10198-021-01424-8)
Supplement: Supplementary file 6 — Supplementary file6 (DOCX 143 KB) [file 10198_2021_1424_MOESM6_ESM.docx]

***Online resource 6***

***Fig. S5 The distribution of potential acceptability of HAcs***
