# Supplementary material for: Measuring the acceptability of EQ-5D-3L health states for different ages: a new adaptive survey methodology
Source: Eur J Health Econ. 2022 Jan 5;23(7):1243–55. doi: 10.1007/s10198-021-01424-8 (PMC9395309; doi:10.1007/s10198-021-01424-8)
Supplement: Supplementary file 7 — Supplementary file7 (DOCX 144 KB) [file 10198_2021_1424_MOESM7_ESM.docx]

***Online resource 7***

***Table S2 Acceptability set: estimated acceptability of EQ-5D health profiles by age***

| EQ-5D-3L | Age: 30 |  | Age: 40 |  | Age: 50 |  | Age: 60 |  | Age: 70 |  | Age: 80 |  |
| --- | --- | --- | --- | --- | --- | --- | --- | --- | --- | --- | --- | --- |
| 11111 | 1.000 |  | 1.000 |  | 1.000 |  | 1.000 |  | 1.000 |  | 1.000 |  |
| 11112 | 0.119 | + | 0.179 | + | 0.272 | + | 0.396 | + | 0.544 | + | 0.659 | + |
| 11113 | 0.049 | + | 0.069 | + | 0.102 | + | 0.158 | + | 0.258 | + | 0.404 | + |
| 11121 | 0.049 | + | 0.105 | + | 0.243 | + | 0.479 | + | 0.746 | + | 0.911 | + |
| 11122 | 0.036 | * | 0.072 | * | 0.165 | * | 0.277 | * | 0.461 | * | 0.605 | * |
| 11123 | 0.016 | * | 0.028 | * | 0.064 | * | 0.114 | * | 0.216 | * | 0.328 | * |
| 11131 | 0.014 | + | 0.024 | + | 0.046 | + | 0.110 | + | 0.258 | + | 0.522 | + |
| 11132 | 0.007 |  | 0.014 |  | 0.029 | * | 0.075 | * | 0.176 | * | 0.345 | * |
| 11133 | 0.007 |  | 0.012 |  | 0.024 |  | 0.059 | * | 0.116 | * | 0.266 | * |
| 11211 | 0.011 | + | 0.019 | + | 0.062 | + | 0.243 | + | 0.602 | + | 0.887 | + |
| 11212 | 0.009 |  | 0.015 | * | 0.039 | * | 0.150 | * | 0.354 | * | 0.563 | * |
| 11213 | 0.006 |  | 0.007 |  | 0.021 | * | 0.081 | * | 0.185 | * | 0.337 | * |
| 11221 | 0.008 |  | 0.015 | * | 0.038 | * | 0.185 | * | 0.478 | * | 0.793 | * |
| 11222 | 0.008 |  | 0.013 |  | 0.033 | * | 0.144 | * | 0.346 | * | 0.553 | * |
| 11223 | 0.006 |  | 0.007 |  | 0.020 | * | 0.076 | * | 0.163 | * | 0.280 | * |
| 11231 | 0.006 |  | 0.007 |  | 0.016 | * | 0.058 | * | 0.166 | * | 0.451 | * |
| 11232 | 0.005 |  | 0.007 |  | 0.015 | * | 0.053 | * | 0.147 | * | 0.317 | * |
| 11233 | 0.005 |  | 0.006 |  | 0.014 |  | 0.045 | * | 0.093 | * | 0.262 | * |
| 11311 | 0.006 | + | 0.008 | + | 0.021 | + | 0.042 | + | 0.141 | + | 0.552 | + |
| 11312 | 0.004 |  | 0.006 |  | 0.013 | * | 0.028 | * | 0.090 | * | 0.420 | * |
| 11313 | 0.004 |  | 0.005 |  | 0.012 |  | 0.023 | * | 0.073 | * | 0.249 | * |
| 11321 | 0.005 |  | 0.006 |  | 0.013 | * | 0.030 | * | 0.104 | * | 0.452 | * |
| 11322 | 0.004 |  | 0.006 |  | 0.012 | * | 0.026 | * | 0.088 | * | 0.323 | * |
| 11323 | 0.004 |  | 0.005 |  | 0.011 |  | 0.021 | * | 0.068 | * | 0.233 | * |
| 11331 | 0.004 |  | 0.005 |  | 0.011 |  | 0.021 | * | 0.070 | * | 0.311 | * |
| 11332 | 0.004 |  | 0.004 |  | 0.010 |  | 0.019 | * | 0.061 | * | 0.255 | * |
| 11333 | 0.003 |  | 0.004 |  | 0.009 |  | 0.017 | * | 0.057 | * | 0.195 | * |
| 12111 | 0.012 | + | 0.017 | + | 0.038 | + | 0.118 | + | 0.388 | + | 0.794 | + |
| 12112 | 0.008 |  | 0.012 |  | 0.027 | * | 0.087 | * | 0.271 | * | 0.554 | * |
| 12113 | 0.006 |  | 0.008 |  | 0.018 | * | 0.061 | * | 0.139 | * | 0.321 | * |
| 12121 | 0.009 |  | 0.012 |  | 0.029 | * | 0.096 | * | 0.340 | * | 0.710 | * |
| 12122 | 0.007 |  | 0.010 |  | 0.025 | * | 0.081 | * | 0.256 | * | 0.529 | * |
| 12123 | 0.006 |  | 0.007 |  | 0.017 | * | 0.057 | * | 0.138 | * | 0.312 | * |
| 12131 | 0.006 |  | 0.007 |  | 0.016 | * | 0.046 | * | 0.138 | * | 0.385 | * |
| 12132 | 0.005 |  | 0.005 |  | 0.014 | * | 0.041 | * | 0.117 | * | 0.297 | * |
| 12133 | 0.004 |  | 0.005 |  | 0.013 |  | 0.036 | * | 0.094 | * | 0.255 | * |
| 12211 | 0.007 |  | 0.010 |  | 0.024 | * | 0.097 | * | 0.332 | * | 0.736 | * |
| 12212 | 0.007 |  | 0.009 |  | 0.020 | * | 0.082 | * | 0.258 | * | 0.563 | * |
| 12213 | 0.005 |  | 0.006 |  | 0.014 | * | 0.056 | * | 0.133 | * | 0.326 | * |

* HAcs with a single health problem

+ 750 HAcs included in the JE frame

| EQ-5D-3L | Age: 30 |  | Age: 40 |  | Age: 50 |  | Age: 60 |  | Age: 70 |  | Age: 80 |  |
| --- | --- | --- | --- | --- | --- | --- | --- | --- | --- | --- | --- | --- |
| 12221 | 0.007 |  | 0.009 |  | 0.022 | * | 0.084 | * | 0.283 | * | 0.688 | * |
| 12222 | 0.006 |  | 0.009 |  | 0.020 | * | 0.076 | * | 0.242 | * | 0.489 | * |
| 12223 | 0.005 |  | 0.006 |  | 0.014 |  | 0.052 | * | 0.133 | * | 0.301 | * |
| 12231 | 0.005 |  | 0.006 |  | 0.013 | * | 0.041 | * | 0.132 | * | 0.372 | * |
| 12232 | 0.004 |  | 0.005 |  | 0.012 |  | 0.038 | * | 0.115 | * | 0.291 | * |
| 12233 | 0.004 |  | 0.004 |  | 0.011 |  | 0.031 | * | 0.089 | * | 0.225 | * |
| 12311 | 0.004 |  | 0.005 |  | 0.011 | * | 0.023 | * | 0.088 | * | 0.433 | * |
| 12312 | 0.003 |  | 0.004 |  | 0.010 | * | 0.021 | * | 0.076 | * | 0.283 | * |
| 12313 | 0.003 |  | 0.004 |  | 0.009 |  | 0.019 | * | 0.067 | * | 0.250 | * |
| 12321 | 0.004 |  | 0.005 |  | 0.010 | * | 0.022 | * | 0.086 | * | 0.450 | * |
| 12322 | 0.003 |  | 0.004 |  | 0.009 |  | 0.020 | * | 0.072 | * | 0.351 | * |
| 12323 | 0.003 |  | 0.004 |  | 0.009 |  | 0.017 | * | 0.068 | * | 0.243 | * |
| 12331 | 0.003 |  | 0.004 |  | 0.009 |  | 0.016 | * | 0.064 | * | 0.263 | * |
| 12332 | 0.003 |  | 0.004 |  | 0.008 |  | 0.015 | * | 0.056 | * | 0.192 | * |
| 12333 | 0.003 |  | 0.003 |  | 0.007 |  | 0.014 | * | 0.050 | * | 0.182 | * |
| 13111 | 0.007 | + | 0.008 | + | 0.015 | + | 0.032 | + | 0.106 | + | 0.468 | + |
| 13112 | 0.004 |  | 0.005 |  | 0.008 |  | 0.021 | * | 0.076 | * | 0.268 | * |
| 13113 | 0.004 |  | 0.005 |  | 0.008 |  | 0.019 | * | 0.068 | * | 0.238 | * |
| 13121 | 0.005 |  | 0.006 |  | 0.009 |  | 0.023 | * | 0.079 | * | 0.373 | * |
| 13122 | 0.004 |  | 0.005 |  | 0.008 |  | 0.019 | * | 0.073 | * | 0.269 | * |
| 13123 | 0.004 |  | 0.004 |  | 0.008 |  | 0.017 | * | 0.065 | * | 0.261 | * |
| 13131 | 0.004 |  | 0.004 |  | 0.008 |  | 0.018 | * | 0.065 | * | 0.274 | * |
| 13132 | 0.003 |  | 0.003 |  | 0.007 |  | 0.016 | * | 0.058 | * | 0.233 | * |
| 13133 | 0.002 |  | 0.003 |  | 0.007 |  | 0.015 | * | 0.052 | * | 0.187 | * |
| 13211 | 0.003 |  | 0.004 |  | 0.010 |  | 0.022 | * | 0.079 | * | 0.407 | * |
| 13212 | 0.003 |  | 0.004 |  | 0.008 |  | 0.020 | * | 0.072 | * | 0.298 | * |
| 13213 | 0.003 |  | 0.004 |  | 0.008 |  | 0.018 | * | 0.064 | * | 0.237 | * |
| 13221 | 0.003 |  | 0.004 |  | 0.008 |  | 0.021 | * | 0.075 | * | 0.329 | * |
| 13222 | 0.003 |  | 0.004 |  | 0.008 |  | 0.018 | * | 0.069 | * | 0.278 | * |
| 13223 | 0.003 |  | 0.004 |  | 0.007 |  | 0.016 | * | 0.062 | * | 0.217 | * |
| 13231 | 0.003 |  | 0.004 |  | 0.007 |  | 0.017 | * | 0.061 | * | 0.277 | * |
| 13232 | 0.002 |  | 0.003 |  | 0.007 |  | 0.015 | * | 0.055 | * | 0.200 | * |
| 13233 | 0.002 |  | 0.003 |  | 0.006 |  | 0.014 | * | 0.049 | * | 0.167 | * |
| 13311 | 0.003 |  | 0.004 |  | 0.008 |  | 0.017 | * | 0.061 | * | 0.295 | * |
| 13312 | 0.003 |  | 0.003 |  | 0.006 |  | 0.015 | * | 0.053 | * | 0.224 | * |
| 13313 | 0.002 |  | 0.003 |  | 0.006 |  | 0.014 | * | 0.048 | * | 0.191 | * |
| 13321 | 0.003 |  | 0.004 |  | 0.006 |  | 0.016 | * | 0.058 | * | 0.279 | * |
| 13322 | 0.002 |  | 0.003 |  | 0.006 |  | 0.014 | * | 0.051 | * | 0.213 | * |
| 13323 | 0.002 |  | 0.003 |  | 0.006 |  | 0.012 | * | 0.045 | * | 0.177 | * |
| 13331 | 0.002 |  | 0.003 |  | 0.006 |  | 0.013 | * | 0.045 | * | 0.218 | * |
| 13332 | 0.002 |  | 0.003 |  | 0.005 |  | 0.011 | * | 0.040 | * | 0.177 | * |
| 13333 | 0.002 |  | 0.003 |  | 0.005 |  | 0.010 |  | 0.036 | * | 0.157 | * |
| 21111 | 0.022 | + | 0.040 | + | 0.153 | + | 0.422 | + | 0.754 | + | 0.933 | + |

* HAcs with a single health problem

+ 750 HAcs included in the JE frame

| EQ-5D-3L | Age: 30 |  | Age: 40 |  | Age: 50 |  | Age: 60 |  | Age: 70 |  | Age: 80 |  |
| --- | --- | --- | --- | --- | --- | --- | --- | --- | --- | --- | --- | --- |
| 21112 | 0.010 |  | 0.020 | * | 0.091 | * | 0.234 | * | 0.437 | * | 0.606 | * |
| 21113 | 0.006 |  | 0.012 |  | 0.042 | * | 0.093 | * | 0.204 | * | 0.315 | * |
| 21121 | 0.010 |  | 0.022 | * | 0.091 | * | 0.297 | * | 0.622 | * | 0.847 | * |
| 21122 | 0.009 |  | 0.018 |  | 0.072 | * | 0.195 | * | 0.396 | * | 0.587 | * |
| 21123 | 0.006 |  | 0.009 |  | 0.035 | * | 0.087 | * | 0.224 | * | 0.309 | * |
| 21131 | 0.004 |  | 0.007 |  | 0.025 | * | 0.072 | * | 0.185 | * | 0.386 | * |
| 21132 | 0.004 |  | 0.007 |  | 0.022 | * | 0.064 | * | 0.160 | * | 0.338 | * |
| 21133 | 0.004 |  | 0.006 |  | 0.018 |  | 0.050 | * | 0.114 | * | 0.255 | * |
| 21211 | 0.006 |  | 0.011 | * | 0.040 | * | 0.186 | * | 0.526 | * | 0.834 | * |
| 21212 | 0.005 |  | 0.009 |  | 0.030 | * | 0.129 | * | 0.320 | * | 0.561 | * |
| 21213 | 0.004 |  | 0.005 |  | 0.018 | * | 0.072 | * | 0.144 | * | 0.287 | * |
| 21221 | 0.006 |  | 0.010 |  | 0.032 | * | 0.180 | * | 0.464 | * | 0.757 | * |
| 21222 | 0.005 |  | 0.009 |  | 0.027 | * | 0.114 | * | 0.338 | * | 0.553 | * |
| 21223 | 0.003 |  | 0.005 |  | 0.017 |  | 0.069 | * | 0.166 | * | 0.317 | * |
| 21231 | 0.003 |  | 0.005 |  | 0.015 | * | 0.053 | * | 0.178 | * | 0.407 | * |
| 21232 | 0.003 |  | 0.005 |  | 0.014 |  | 0.049 | * | 0.140 | * | 0.285 | * |
| 21233 | 0.003 |  | 0.004 |  | 0.013 |  | 0.041 | * | 0.117 | * | 0.231 | * |
| 21311 | 0.003 |  | 0.005 |  | 0.014 | * | 0.029 | * | 0.105 | * | 0.449 | * |
| 21312 | 0.003 |  | 0.004 |  | 0.012 | * | 0.025 | * | 0.083 | * | 0.320 | * |
| 21313 | 0.003 |  | 0.004 |  | 0.010 |  | 0.020 | * | 0.067 | * | 0.248 | * |
| 21321 | 0.003 |  | 0.005 |  | 0.012 | * | 0.027 | * | 0.092 | * | 0.418 | * |
| 21322 | 0.003 |  | 0.004 |  | 0.011 |  | 0.023 | * | 0.079 | * | 0.321 | * |
| 21323 | 0.003 |  | 0.004 |  | 0.010 |  | 0.019 | * | 0.064 | * | 0.229 | * |
| 21331 | 0.003 |  | 0.004 |  | 0.009 |  | 0.018 | * | 0.065 | * | 0.302 | * |
| 21332 | 0.002 |  | 0.003 |  | 0.009 |  | 0.016 | * | 0.059 | * | 0.215 | * |
| 21333 | 0.002 |  | 0.003 |  | 0.008 |  | 0.015 | * | 0.053 | * | 0.184 | * |
| 22111 | 0.006 |  | 0.009 |  | 0.027 | * | 0.096 | * | 0.332 | * | 0.754 | * |
| 22112 | 0.005 |  | 0.008 |  | 0.022 | * | 0.083 | * | 0.259 | * | 0.526 | * |
| 22113 | 0.004 |  | 0.005 |  | 0.015 | * | 0.056 | * | 0.136 | * | 0.340 | * |
| 22121 | 0.005 |  | 0.008 |  | 0.024 | * | 0.087 | * | 0.301 | * | 0.700 | * |
| 22122 | 0.005 |  | 0.007 |  | 0.021 | * | 0.077 | * | 0.242 | * | 0.505 | * |
| 22123 | 0.003 |  | 0.004 |  | 0.014 |  | 0.052 | * | 0.145 | * | 0.277 | * |
| 22131 | 0.003 |  | 0.004 |  | 0.013 | * | 0.044 | * | 0.131 | * | 0.362 | * |
| 22132 | 0.002 |  | 0.003 |  | 0.012 |  | 0.039 | * | 0.116 | * | 0.299 | * |
| 22133 | 0.002 |  | 0.003 |  | 0.011 |  | 0.034 | * | 0.089 | * | 0.235 | * |
| 22211 | 0.004 |  | 0.006 |  | 0.022 | * | 0.087 | * | 0.287 | * | 0.685 | * |
| 22212 | 0.004 |  | 0.006 |  | 0.019 | * | 0.076 | * | 0.246 | * | 0.545 | * |
| 22213 | 0.003 |  | 0.004 |  | 0.013 |  | 0.051 | * | 0.128 | * | 0.283 | * |
| 22221 | 0.004 |  | 0.006 |  | 0.020 | * | 0.085 | * | 0.293 | * | 0.650 | * |
| 22222 | 0.004 |  | 0.006 |  | 0.018 | * | 0.070 | * | 0.212 | * | 0.437 | * |
| 22223 | 0.003 |  | 0.003 |  | 0.012 |  | 0.048 | * | 0.127 | * | 0.306 | * |
| 22231 | 0.003 |  | 0.003 |  | 0.011 |  | 0.039 | * | 0.129 | * | 0.365 | * |
| 22232 | 0.002 |  | 0.003 |  | 0.011 |  | 0.036 | * | 0.104 | * | 0.336 | * |

* HAcs with a single health problem

+ 750 HAcs included in the JE frame

| EQ-5D-3L | Age: 30 |  | Age: 40 |  | Age: 50 |  | Age: 60 |  | Age: 70 |  | Age: 80 |  |
| --- | --- | --- | --- | --- | --- | --- | --- | --- | --- | --- | --- | --- |
| 22233 | 0.002 |  | 0.003 |  | 0.010 |  | 0.029 | * | 0.084 | * | 0.217 | * |
| 22311 | 0.002 |  | 0.003 |  | 0.010 | * | 0.022 | * | 0.091 | * | 0.435 | * |
| 22312 | 0.002 |  | 0.003 |  | 0.009 |  | 0.020 | * | 0.070 | * | 0.321 | * |
| 22313 | 0.002 |  | 0.003 |  | 0.008 |  | 0.017 | * | 0.065 | * | 0.225 | * |
| 22321 | 0.002 |  | 0.003 |  | 0.009 |  | 0.020 | * | 0.079 | * | 0.400 | * |
| 22322 | 0.002 |  | 0.003 |  | 0.008 |  | 0.018 | * | 0.067 | * | 0.287 | * |
| 22323 | 0.002 |  | 0.003 |  | 0.008 |  | 0.016 | * | 0.060 | * | 0.188 | * |
| 22331 | 0.002 |  | 0.003 |  | 0.008 |  | 0.015 | * | 0.060 | * | 0.264 | * |
| 22332 | 0.002 |  | 0.003 |  | 0.007 |  | 0.014 | * | 0.052 | * | 0.189 | * |
| 22333 | 0.002 |  | 0.002 |  | 0.007 |  | 0.012 |  | 0.046 | * | 0.161 | * |
| 23111 | 0.003 |  | 0.005 |  | 0.010 |  | 0.022 | * | 0.073 | * | 0.392 | * |
| 23112 | 0.003 |  | 0.004 |  | 0.007 |  | 0.019 | * | 0.071 | * | 0.277 | * |
| 23113 | 0.003 |  | 0.004 |  | 0.007 |  | 0.017 | * | 0.064 | * | 0.222 | * |
| 23121 | 0.003 |  | 0.004 |  | 0.008 |  | 0.021 | * | 0.074 | * | 0.361 | * |
| 23122 | 0.003 |  | 0.004 |  | 0.007 |  | 0.018 | * | 0.069 | * | 0.273 | * |
| 23123 | 0.003 |  | 0.004 |  | 0.007 |  | 0.016 | * | 0.061 | * | 0.197 | * |
| 23131 | 0.002 |  | 0.003 |  | 0.007 |  | 0.017 | * | 0.061 | * | 0.251 | * |
| 23132 | 0.002 |  | 0.003 |  | 0.006 |  | 0.015 | * | 0.055 | * | 0.213 | * |
| 23133 | 0.002 |  | 0.002 |  | 0.006 |  | 0.014 | * | 0.049 | * | 0.145 | * |
| 23211 | 0.002 |  | 0.003 |  | 0.008 |  | 0.021 | * | 0.074 | * | 0.354 | * |
| 23212 | 0.002 |  | 0.003 |  | 0.007 |  | 0.018 | * | 0.068 | * | 0.291 | * |
| 23213 | 0.002 |  | 0.003 |  | 0.007 |  | 0.017 | * | 0.060 | * | 0.224 | * |
| 23221 | 0.002 |  | 0.003 |  | 0.007 |  | 0.019 | * | 0.070 | * | 0.326 | * |
| 23222 | 0.002 |  | 0.003 |  | 0.007 |  | 0.017 | * | 0.065 | * | 0.286 | * |
| 23223 | 0.002 |  | 0.003 |  | 0.006 |  | 0.015 | * | 0.058 | * | 0.178 | * |
| 23231 | 0.002 |  | 0.003 |  | 0.006 |  | 0.016 | * | 0.058 | * | 0.232 | * |
| 23232 | 0.002 |  | 0.003 |  | 0.006 |  | 0.014 | * | 0.051 | * | 0.231 | * |
| 23233 | 0.002 |  | 0.002 |  | 0.005 |  | 0.013 |  | 0.046 | * | 0.184 | * |
| 23311 | 0.002 |  | 0.003 |  | 0.007 |  | 0.016 | * | 0.057 | * | 0.281 | * |
| 23312 | 0.002 |  | 0.003 |  | 0.005 |  | 0.014 | * | 0.050 | * | 0.270 | * |
| 23313 | 0.002 |  | 0.002 |  | 0.005 |  | 0.013 | * | 0.045 | * | 0.191 | * |
| 23321 | 0.002 |  | 0.003 |  | 0.006 |  | 0.015 | * | 0.054 | * | 0.281 | * |
| 23322 | 0.002 |  | 0.002 |  | 0.005 |  | 0.013 | * | 0.047 | * | 0.220 | * |
| 23323 | 0.002 |  | 0.002 |  | 0.005 |  | 0.011 |  | 0.042 | * | 0.164 | * |
| 23331 | 0.002 |  | 0.002 |  | 0.005 |  | 0.012 | * | 0.042 | * | 0.214 | * |
| 23332 | 0.001 |  | 0.002 |  | 0.004 |  | 0.010 |  | 0.037 | * | 0.165 | * |
| 23333 | 0.001 |  | 0.002 |  | 0.004 |  | 0.009 |  | 0.033 | * | 0.138 | * |
| 31111 | 0.008 | + | 0.009 | + | 0.020 | + | 0.039 | + | 0.120 | + | 0.424 | + |
| 31112 | 0.004 |  | 0.005 |  | 0.011 | * | 0.025 | * | 0.078 | * | 0.259 | * |
| 31113 | 0.004 |  | 0.004 |  | 0.010 |  | 0.020 | * | 0.069 | * | 0.195 | * |
| 31121 | 0.004 |  | 0.005 |  | 0.011 | * | 0.026 | * | 0.080 | * | 0.316 | * |
| 31122 | 0.004 |  | 0.004 |  | 0.010 | * | 0.022 | * | 0.073 | * | 0.218 | * |
| 31123 | 0.004 |  | 0.004 |  | 0.009 |  | 0.019 | * | 0.065 | * | 0.206 | * |

* HAcs with a single health problem

+ 750 HAcs included in the JE frame

| EQ-5D-3L | Age: 30 |  | Age: 40 |  | Age: 50 |  | Age: 60 |  | Age: 70 |  | Age: 80 |  |
| --- | --- | --- | --- | --- | --- | --- | --- | --- | --- | --- | --- | --- |
| 31131 | 0.003 |  | 0.004 |  | 0.008 |  | 0.019 | * | 0.066 | * | 0.251 | * |
| 31132 | 0.003 |  | 0.004 |  | 0.007 |  | 0.017 | * | 0.058 | * | 0.200 | * |
| 31133 | 0.003 |  | 0.004 |  | 0.007 |  | 0.015 | * | 0.054 | * | 0.179 | * |
| 31211 | 0.003 |  | 0.004 |  | 0.009 | * | 0.024 | * | 0.083 | * | 0.351 | * |
| 31212 | 0.003 |  | 0.004 |  | 0.007 | * | 0.022 | * | 0.073 | * | 0.257 | * |
| 31213 | 0.003 |  | 0.004 |  | 0.007 |  | 0.019 | * | 0.064 | * | 0.200 | * |
| 31221 | 0.003 |  | 0.004 |  | 0.008 | * | 0.023 | * | 0.078 | * | 0.303 | * |
| 31222 | 0.003 |  | 0.004 |  | 0.007 |  | 0.020 | * | 0.069 | * | 0.243 | * |
| 31223 | 0.002 |  | 0.004 |  | 0.007 |  | 0.017 | * | 0.061 | * | 0.171 | * |
| 31231 | 0.002 |  | 0.004 |  | 0.006 |  | 0.017 | * | 0.061 | * | 0.232 | * |
| 31232 | 0.002 |  | 0.003 |  | 0.006 |  | 0.015 | * | 0.057 | * | 0.184 | * |
| 31233 | 0.002 |  | 0.003 |  | 0.005 |  | 0.014 | * | 0.050 | * | 0.147 | * |
| 31311 | 0.003 |  | 0.003 |  | 0.007 |  | 0.015 | * | 0.051 | * | 0.276 | * |
| 31312 | 0.002 |  | 0.003 |  | 0.006 |  | 0.013 | * | 0.045 | * | 0.211 | * |
| 31313 | 0.002 |  | 0.003 |  | 0.006 |  | 0.012 | * | 0.039 | * | 0.163 | * |
| 31321 | 0.002 |  | 0.003 |  | 0.006 |  | 0.014 | * | 0.048 | * | 0.231 | * |
| 31322 | 0.002 |  | 0.003 |  | 0.006 |  | 0.012 | * | 0.042 | * | 0.174 | * |
| 31323 | 0.002 |  | 0.003 |  | 0.005 |  | 0.011 | * | 0.037 | * | 0.170 | * |
| 31331 | 0.002 |  | 0.003 |  | 0.005 |  | 0.011 | * | 0.039 | * | 0.190 | * |
| 31332 | 0.002 |  | 0.002 |  | 0.004 |  | 0.010 | * | 0.034 | * | 0.156 | * |
| 31333 | 0.002 |  | 0.002 |  | 0.004 |  | 0.009 |  | 0.030 | * | 0.124 | * |
| 32111 | 0.002 |  | 0.003 |  | 0.009 | * | 0.023 | * | 0.076 | * | 0.319 | * |
| 32112 | 0.002 |  | 0.003 |  | 0.006 | * | 0.019 | * | 0.073 | * | 0.248 | * |
| 32113 | 0.002 |  | 0.003 |  | 0.006 |  | 0.016 | * | 0.065 | * | 0.186 | * |
| 32121 | 0.002 |  | 0.003 |  | 0.007 | * | 0.021 | * | 0.077 | * | 0.317 | * |
| 32122 | 0.002 |  | 0.003 |  | 0.006 |  | 0.018 | * | 0.064 | * | 0.205 | * |
| 32123 | 0.002 |  | 0.002 |  | 0.006 |  | 0.015 | * | 0.061 | * | 0.187 | * |
| 32131 | 0.002 |  | 0.002 |  | 0.006 |  | 0.016 | * | 0.061 | * | 0.227 | * |
| 32132 | 0.002 |  | 0.002 |  | 0.005 |  | 0.014 | * | 0.057 | * | 0.188 | * |
| 32133 | 0.002 |  | 0.002 |  | 0.005 |  | 0.012 | * | 0.050 | * | 0.160 | * |
| 32211 | 0.002 |  | 0.003 |  | 0.007 | * | 0.021 | * | 0.078 | * | 0.280 | * |
| 32212 | 0.002 |  | 0.003 |  | 0.006 |  | 0.019 | * | 0.068 | * | 0.249 | * |
| 32213 | 0.002 |  | 0.002 |  | 0.005 |  | 0.016 | * | 0.061 | * | 0.170 | * |
| 32221 | 0.002 |  | 0.003 |  | 0.006 |  | 0.019 | * | 0.072 | * | 0.298 | * |
| 32222 | 0.002 |  | 0.003 |  | 0.005 |  | 0.017 | * | 0.065 | * | 0.252 | * |
| 32223 | 0.002 |  | 0.002 |  | 0.005 |  | 0.014 | * | 0.058 | * | 0.177 | * |
| 32231 | 0.002 |  | 0.002 |  | 0.005 |  | 0.014 | * | 0.058 | * | 0.199 | * |
| 32232 | 0.002 |  | 0.002 |  | 0.005 |  | 0.013 | * | 0.054 | * | 0.198 | * |
| 32233 | 0.002 |  | 0.002 |  | 0.004 |  | 0.012 |  | 0.047 | * | 0.184 | * |
| 32311 | 0.002 |  | 0.002 |  | 0.006 |  | 0.013 | * | 0.049 | * | 0.265 | * |
| 32312 | 0.002 |  | 0.002 |  | 0.005 |  | 0.012 | * | 0.042 | * | 0.192 | * |
| 32313 | 0.002 |  | 0.002 |  | 0.005 |  | 0.011 | * | 0.038 | * | 0.144 | * |
| 32321 | 0.002 |  | 0.002 |  | 0.005 |  | 0.012 | * | 0.046 | * | 0.222 | * |
| 32322 | 0.002 |  | 0.002 |  | 0.005 |  | 0.011 | * | 0.040 | * | 0.173 | * |

* HAcs with a single health problem

+ 750 HAcs included in the JE frame

| EQ-5D-3L | Age: 30 |  | Age: 40 |  | Age: 50 |  | Age: 60 |  | Age: 70 |  | Age: 80 |  |
| --- | --- | --- | --- | --- | --- | --- | --- | --- | --- | --- | --- | --- |
| 32323 | 0.002 |  | 0.002 |  | 0.004 |  | 0.009 |  | 0.036 | * | 0.160 | * |
| 32331 | 0.002 |  | 0.002 |  | 0.004 |  | 0.009 | * | 0.037 | * | 0.205 | * |
| 32332 | 0.001 |  | 0.002 |  | 0.004 |  | 0.008 |  | 0.032 | * | 0.163 | * |
| 32333 | 0.001 |  | 0.002 |  | 0.003 |  | 0.008 |  | 0.028 | * | 0.118 | * |
| 33111 | 0.002 |  | 0.003 |  | 0.007 |  | 0.014 | * | 0.057 | * | 0.232 | * |
| 33112 | 0.002 |  | 0.002 |  | 0.005 |  | 0.012 | * | 0.050 | * | 0.209 | * |
| 33113 | 0.002 |  | 0.002 |  | 0.004 |  | 0.011 | * | 0.045 | * | 0.185 | * |
| 33121 | 0.002 |  | 0.003 |  | 0.005 |  | 0.013 | * | 0.054 | * | 0.230 | * |
| 33122 | 0.002 |  | 0.002 |  | 0.004 |  | 0.011 | * | 0.048 | * | 0.172 | * |
| 33123 | 0.002 |  | 0.002 |  | 0.004 |  | 0.010 | * | 0.043 | * | 0.167 | * |
| 33131 | 0.002 |  | 0.002 |  | 0.005 |  | 0.010 | * | 0.043 | * | 0.191 | * |
| 33132 | 0.001 |  | 0.002 |  | 0.004 |  | 0.009 | * | 0.038 | * | 0.138 | * |
| 33133 | 0.001 |  | 0.002 |  | 0.003 |  | 0.008 |  | 0.034 | * | 0.151 | * |
| 33211 | 0.002 |  | 0.002 |  | 0.005 |  | 0.013 | * | 0.053 | * | 0.222 | * |
| 33212 | 0.002 |  | 0.002 |  | 0.004 |  | 0.012 | * | 0.047 | * | 0.185 | * |
| 33213 | 0.002 |  | 0.002 |  | 0.004 |  | 0.010 | * | 0.042 | * | 0.141 | * |
| 33221 | 0.002 |  | 0.002 |  | 0.004 |  | 0.012 | * | 0.050 | * | 0.268 | * |
| 33222 | 0.002 |  | 0.002 |  | 0.004 |  | 0.010 | * | 0.045 | * | 0.181 | * |
| 33223 | 0.002 |  | 0.002 |  | 0.004 |  | 0.009 |  | 0.040 | * | 0.163 | * |
| 33231 | 0.002 |  | 0.002 |  | 0.004 |  | 0.009 | * | 0.040 | * | 0.178 | * |
| 33232 | 0.001 |  | 0.002 |  | 0.003 |  | 0.008 |  | 0.035 | * | 0.140 | * |
| 33233 | 0.001 |  | 0.002 |  | 0.003 |  | 0.007 |  | 0.031 | * | 0.106 | * |
| 33311 | 0.002 |  | 0.002 |  | 0.004 |  | 0.011 | * | 0.039 | * | 0.182 | * |
| 33312 | 0.001 |  | 0.002 |  | 0.004 |  | 0.010 | * | 0.034 | * | 0.172 | * |
| 33313 | 0.001 |  | 0.002 |  | 0.003 |  | 0.008 |  | 0.030 | * | 0.116 | * |
| 33321 | 0.002 |  | 0.002 |  | 0.004 |  | 0.010 | * | 0.037 | * | 0.198 | * |
| 33322 | 0.001 |  | 0.002 |  | 0.003 |  | 0.009 |  | 0.032 | * | 0.157 | * |
| 33323 | 0.001 |  | 0.002 |  | 0.003 |  | 0.007 |  | 0.028 | * | 0.093 | * |
| 33331 | 0.001 |  | 0.002 |  | 0.003 |  | 0.007 |  | 0.029 | * | 0.151 | * |
| 33332 | 0.001 |  | 0.001 |  | 0.003 |  | 0.006 |  | 0.024 | * | 0.107 | * |
| 33333 | 0.001 |  | 0.001 |  | 0.002 |  | 0.006 |  | 0.021 | * | 0.074 | * |

* HAcs with a single health problem

+ 750 HAcs included in the JE frame
